# The effect of transverse wavefront width on specular neutron reflection

**DOI:** 10.1107/S160057672200440X

**Published:** 2022-06-23

**Authors:** C. F. Majkrzak, N. F. Berk, B. B. Maranville, J. A. Dura, T. Jach

**Affiliations:** a Center for Neutron Research, National Institute of Standards and Technology, 100 Bureau Drive, Gaithersburg, MD 20899, USA; b Materials Measurement Laboratory, National Institute of Standards and Technology, 100 Bureau Drive, Gaithersburg, MD 20899, USA; Australian Centre for Neutron Scattering, ANSTO, Australia

**Keywords:** specular neutron reflectivity, wave packets, transverse coherent extent

## Abstract

In the analysis of neutron scattering measurements of condensed matter structure, it normally suffices to treat the incident and scattered neutron beams as if composed of incoherent distributions of plane waves with wavevectors of different magnitudes and directions that are taken to define an instrumental resolution. However, despite the wide-ranging applicability of this conventional treatment, there are cases, such as specular neutron reflectometry, in which the structural length scales of the scattering object require that the wavefunction of an individual neutron in the beam be described by a spatially localized packet – in particular with respect to the transverse extent of its wavefronts (*i.e.* normal to the packet’s mean direction of propagation). It is shown in the present work that neutron diffraction patterns observed for periodic transmission phase gratings, as well as specular reflection measurements from patterned thin films with repeat units of the order of micrometres, can be accurately described by associating an individual neutron with a wave packet and treating a beam as a collection of independent packets. In these cases, accurate analysis requires that the transverse spatial extent of a neutron packet wavefront be accounted for in addition to the angular divergence of the beam that is characterized by a distribution of packet mean wavevector directions. It is shown how a measure of the effective transverse spatial extent of the neutron packet – over which its wavefronts are of sufficient uniformity to produce coherent scattering – can be determined by employing reference diffraction gratings and patterned thin films of known structure and composition.

## Abstract 2

The role that the transverse coherent extent of a neutron packet wavefunction plays in the analysis of specular neutron reflectivity is examined both theoretically and experimentally. It is shown how, in practice, the transverse coherent extent of an individual neutron packet wavefront can be measured and distinguished from the effects of geometrical angular divergence for a beam composed of a collection of such independent packets.

## 1. Introduction

Information about the compositional depth profile of a layered thin-film structure can be obtained by measuring the specular reflection of neutrons for which the momentum transfer is normal to the surface or interfaces. Such measurements have proven to be valuable in structural studies of both hard and soft condensed matter, including materials of interest in physics, chemistry and biology. Nonetheless, in the specular condition this profile represents, as a function of depth, a material density averaged across a certain area in a plane perpendicular to the surface normal. If an inhomogeneous density distribution exists in plane, not only will off-specular scattering occur concurrently but a full analysis of the specularly reflected component will require a knowledge of (1) the degree to which the phase across a given area of an incident neutron wavefront is uniform and (2) the nature of the in-plane inhomogeneous density distribution – including the different types and sizes of material regions or domains in comparison to the size of the projected neutron wavefront. As a consequence, the treatment of an incident neutron wavefunction as a plane wave possessing constant phase over an infinite lateral extent is not necessarily a valid approximation.

The purpose of this paper is to clarify the distinct roles that the transverse spatial extent of an individual neutron wavefunction and the angular divergence of a beam composed of such independent neutrons play in neutron reflectometry. In the following sections, we characterize the neutron in a wave packet picture. We consider how, for example, instrument components such as apertures can simultaneously define the angular distribution of neutron packet mean wavevectors in a beam (geometrical collimation) and shape individual neutron wavefunctions (in particular the transverse coherent extent of a packet wavefront). The problem of determining and accounting for the effective transverse coherent extent of a neutron wavefront – specifically the area over which the wavefront is of sufficient uniformity to give rise to coherent reflected waves – is then addressed. (The role of longitudinal coherence, as defined by the fractional uncertainty in the magnitudes of packet wavevectors, is of secondary importance to the specular reflectivity processes considered herein and will only be mentioned peripherally.)

We acknowledge at the outset the pioneering work of Zeilinger, Treimer and others (*e.g.* Zeilinger *et al.*, 1988 ▸; Treimer *et al.*, 2006 ▸), which clearly demonstrated the importance of the coherent transverse extent of a neutron wavefront in diffraction measurements. For example, in single- and double-slit measurements performed by Zeilinger *et al.* (1988 ▸), a special instrumental design employed a prism and slit apertures at sufficient distance to prepare a relatively planar wavefront simultaneously with the creation of an extraordinarily narrow beam angular divergence – equivalent to contracting an extended source width almost to a point. In these experiments, coherent transverse wavefront widths of 100 µm or more were obtained. One of the principal objectives of the work we report here is to show how packet wavefront widths can be empirically determined for more general instrumental configurations: that is, for those instrumental arrangements in which the incoherent distribution or collection of packet mean wavevectors present in a beam – associated with a geometrical angular divergence – is comparable in size to and coexists with each of the coherent distributions of basis wavevectors contained in every individual neutron wave packet in that beam. We treat the effects of the distribution of packet mean wavevectors in a beam composed of a collection of independent packets separately from those which depend on the size and shape of an individual packet.

However, the shaping of a wave packet is a relatively complicated process. Instrumental components such as mirrors, slit apertures and monochromating crystals all contribute in unison to the form of the packet incident on a sample being probed. Moreover, the original form of the packet at its initial point of emission from a source is not necessarily known. Thus, determining the detailed shape and extent of a packet wavefront over which the phase is sufficiently uniform to a specified degree can be difficult if not practically impossible. Nonetheless, it is possible to obtain a reasonable picture of the extent of packet spatial localization to be expected through calculations that model the effect of various instrumental elements, such as a slit aperture or a crystal. Moreover, a practically useful measure of an effective coherent transverse extent of a wavefront – over which the phase is of sufficient uniformity to give rise to a coherently scattered wave from a particular volume of a scattering object – can be obtained empirically, through measurements that employ known reference objects such as periodic diffraction gratings or other patterned film structures.

In this approach, to directly measure the effective transverse width of a neutron wave packet through the use of reference gratings or patterned structures, the aim is not necessarily to characterize a particular piece of graphite or other specific instrumental component. Rather, the objective is to demonstrate and develop a general procedure for determining the transverse spatial extent of a packet produced by the collective action of all of the components of any given instrument.

We begin in Section 2 with a brief description of those aspects of specular reflection which are pertinent to discussions of packet transverse width in subsequent sections. Section 3 gives a basic definition of a ‘free’ incident neutron wave packet, introducing the notion of a beam of such individual packets, and then a simple two-dimensional model for a single wave packet. In Section 4, model calculations are described which predict the packet width created in the process of a neutron interacting with a typical slit aperture or a microcrystalline block of a monochromator such as pyrolytic graphite.

In Section 5, a measurement is described which demonstrates the combined effects of an actual mosaic crystal monochromator and a pair of slits in defining both geometrical beam angular divergence and packet transverse width.

In Section 6, experimental approaches for determining the transverse extent of a packet wavefront are examined. For one method in particular, involving specular reflection from patterned films, the effect of transverse wavefront width is shown to be largely decoupled from beam angular resolution.

In Section 7, we consider a well known theory of partial coherence developed for light optics, with references to well known textbook treatments, with the aim of clarifying the sometimes confusing issue regarding the differences between plane-wave beams and beams of wave packets.

Section 8 very briefly places the earlier sections in a wider space of standard quantum mechanics and is followed by the *Conclusion* [Sec sec9]. Finally, there are two appendices. Appendix *A* [App appa] provides a description of the configuration of the neutron reflectometer at the NIST Center for Neutron Research (NCNR) on which measurements described in other sections were performed. Appendix *B* [App appb] provides a detailed working out of the mathematics appropriate to the different source types discussed in Section 7.

## 2. Specular reflection

The elastic, coherent, specular reflection of neutrons from layered thin-film systems provides information about the scattering length density (SLD) depth profile along the mean surface normal, parallel to the wavevector transfer **Q** = **k**_I_ − **k**_F_, where **k**_I_ and **k**_F_ are the mean wavevectors of the incident and reflected neutrons, respectively. In principle, the reflected neutron wave amplitude is related to the SLD distribution normal to the surface by a Fourier transform if the scattering is sufficiently weak (Born approximation) or through the solution of the Schrödinger equation if the interaction is stronger. In either case, the relationship is taken to be one dimensional insofar as the SLD can be treated as varying only along the surface normal. This condition naturally applies to any system that is perfectly homogeneous along any direction perpendicular to **Q** – designated as being ‘in plane’ – but can also be well approximated by systems possessing in-plane density variations which are sufficiently small that any non-specular reflection is negligible. In the latter case for which in-plane SLD variations do exist, the SLD obtained at a given position along the normal through specular reflection measurements corresponds to the in-plane average at that point.

In the Born approximation for specular reflection, where both the incident and reflected neutron wavefunctions are taken to be plane waves, the reflection amplitude *r*_BA_(*Q*) [the measurable reflectivity is given by |*r*_BA_(*Q*)|^2^] can be written as

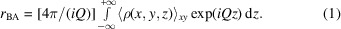

ρ is the SLD, *Q* is the wavevector transfer along the sample normal (which is taken to be parallel to the *z* axis) and

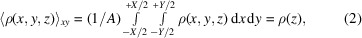

where *A* = *XY* is the area over which an incident neutron wavefront interacts. It can be rigorously shown that this coherent averaging applies to scattering described by exact solutions of the one-dimensional Schrödinger wave equation as well. (As a related example, it is also straightforward to show that, for a given *hkl* reflection from a periodic crystal structure, each of the atomic planes contributing to that reflection – all of which are perpendicular to the corresponding reciprocal lattice vector and wavevector transfer – has an effective scattering density proportional to the average scattering amplitude of all the atoms lying in that particular atomic plane.) Note that, in both the Born approximation and the Schrödinger wave equation formalism, the neutron wavefunctions are typically taken to be plane waves of infinite spatial extent.

In practice, the theoretical representation of a single neutron wavefunction as an ideal plane wave does not allow for a satisfactory treatment of specular reflection in all instances. One problematic case in particular involves specular reflection from certain types of surfaces or interfaces possessing in-plane inhomogeneities. A more accurate description of a freely propagating neutron is that of a quantum particle associated with a spatially localized wave packet possessing wavefronts of finite transverse extent, as will be considered in subsequent sections below. Consequently, a full analysis of specular reflection crucially depends on the length scale of any in-plane material SLD variations which might exist in relation to the actual transverse coherent extent of the neutron wavefront.

**Figure 1:**
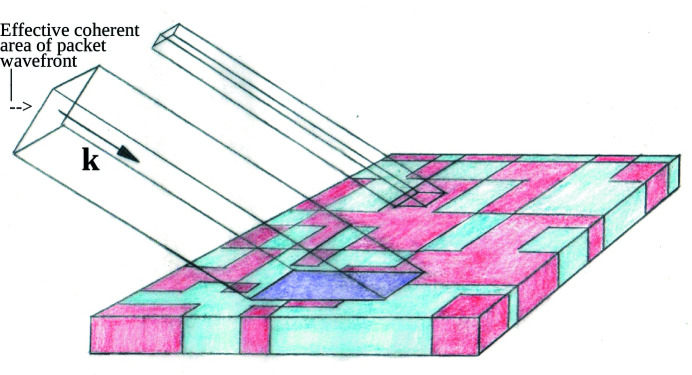
A simple example of an inhomogeneous in-plane density distribution that is made up of only two distinct types of homogeneous region or domain, each type having one of two different values of average in-plane SLD, either ρ_A1_ or ρ_A2_, as discussed in the main body of text. Incident neutron wavefronts of two different transverse sizes projected onto the material surface are shown.

As a simple example, consider an inhomogeneous in-plane density distribution that is made up of only two distinct types of homogeneous regions or domains, each type having one of two different values of in-plane SLD, say ρ_A1_ and ρ_A2_. We assume that the reflecting material surfaces as well as the neutron wavefronts are perfectly flat. Then the finite transverse area of an incident neutron wavefront may or may not be projected onto both types of material regions simultaneously, depending on the relative sizes of wavefront and domains – as is schematically illustrated in Fig. 1 ▸. If the projected wavefront area over which the phase is sufficiently uniform to give rise to a coherently reflected wave is smaller than one of the two different types of regions and is incident entirely within that region, then the contribution to the reflected wave amplitude will be determined by the corresponding value of SLD, *i.e.* either ρ_A1_ or ρ_A2_. In the limiting case where the projected transverse area of the incident neutron wavefront is much smaller than the area of either material region ‘A1’ or ‘A2’, the resultant specular reflectivity |*r*(*Q*)|^2^ is given by

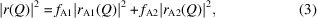

where *f*_A1_ and *f*_A2_ are the fractional material areas with associated reflectivities |*r*_A1_(*Q*)|^2^ and |*r*_A2_(*Q*)|^2^, respectively. On the other hand, if the neutron wavefront is projected over two or more material regions, the contribution to the specularly reflected wave amplitude will correspond to the weighted-area average (as covered by the projected wavefront) of the two different in-plane values of SLD,

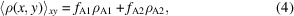

from which the specular reflectivity would then be computed. Clearly, these two different limiting cases correspond to completely different relationships between reflectivity and the scattering length density distribution of the material system. In intermediate cases, depending upon how much is known about the in-plane density distribution, it may not be possible to interpret the specular reflection data accurately. It is therefore essential to know the relevant length scales of neutron wavefront and domain sizes of any inhomogeneous in-plane material density distributions in order to analyze reflectivity data properly. [Another specific instance in which the spatial size of the neutron wave packet matters pertains to the formation of orbital angular momentum (OAM) states of the neutron. The transverse extent over which the phase of a neutron packet wavefront is of the requisite uniformity determines whether or not a suitably structured material object of a given size can impart OAM to a neutron (Cappelletti *et al.*, 2018 ▸).]

## 3. A more general description of a neutron beam

For the analysis of a broad range of neutron scattering data involving studies of condensed matter structure, it suffices to treat the incident and scattered neutron beams as if composed of non-interfering incoherent distributions of plane waves with wavevectors of different magnitudes and directions. These distributions (typically assumed to be Gaussian) are conventionally taken to correspond to an instrumental resolution. Nonetheless, in certain circumstances – in particular that of specular reflection from surfaces or interfaces possessing in-plane density distributions as described in the preceding section[Sec sec2] – a description that accounts for neutron wavefronts of finite width is necessary.

In reality, an incident neutron beam employed in a typical reflectometer is more accurately described as an ensemble of independent, non-interacting and freely propagating quantum particles, each of which can be associated with a spatially localized wavefunction or ‘packet’. This spatial localization is, in general, in directions both transverse and parallel to the packet’s mean propagation direction. (The packet also possesses a temporal localization, although this is not of direct relevance in the present work.) The packet contains wavefronts of finite transverse extent even though mathematically it can be constructed, for example, from a coherent superposition of planar basis states. The wave packet description for freely propagating fermions is supported by observation, perhaps most convincingly by slit diffraction measurements with electrons at exceedingly low intensities (Tonomura *et al.*, 1989 ▸). Even when a single diffracted electron is detected at a time, the characteristic interference pattern emerges after a statistically meaningful number of events are recorded. [For the diffraction grating measurements performed with highly collimated and monochromatic neutrons reported in this work (described in Section 6), at a nominal wavelength of 5 Å, a free neutron travels 791 m in 1 s. Thus, at a typical detection rate of one per second, the average spacing between consecutive neutrons in the beam is 791 m. Since the path length within the instrument is only approximately 2 m, there is most often only a single neutron within the instrument at a time. But even if that were not the case, the cold neutrons in the beam are independent and non-interacting with one another to an exceedingly good approximation.] Moreover, according to standard quantum theory, there is rigorous justification for describing a single neutron packet wavefunction as a pure state, whereas a beam would then be composed of an ensemble of individual neutron packets as a mixed state (*e.g.* Cohen-Tannoudji *et al.*, 1977 ▸; Merzbacher, 1961 ▸; Berk, 2014 ▸, 2018 ▸). The subsequent interaction of each neutron in the beam with a condensed matter structure is then described by the evolution of its associated wave packet according to the time-dependent Schrödinger equation of motion. Each neutron in the beam is treated separately, one at a time, in a manner first emphasized by Dirac (1958 ▸). (On the other hand, for light scattering, Maxwell’s equations for both electric and magnetic fields need to be solved, requiring the introduction of many-body photon states to describe the light produced, for example, by a laser source.)

In summary, the wave packet describing the state of an isolated neutron can be represented by a coherent superposition of weighted plane wavefunctions. Moreover, a beam of non-interacting neutrons can, in most practical cases, be associated with a corresponding set of isolated wave packets. In other words, a beam is formed from an incoherent (non-interfering) ensemble of such isolated packets and is characterized by a distribution of the directions (and, in general, also magnitudes) of the individual packet mean wavevectors. In this particular description, the ‘system’ is represented by a single neutron interacting with a material object. Employing a beam, on the other hand, corresponds to the repetition of such single-neutron scattering experiments over a range of different incident directions a statistically significant number of times.

**Figure 2:**
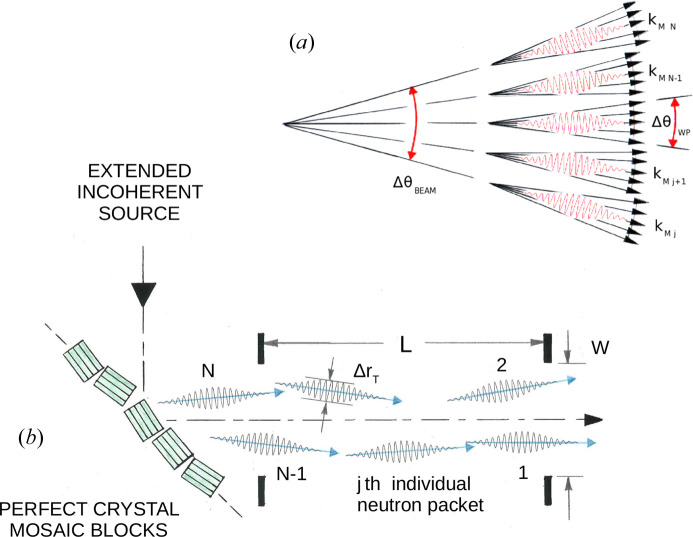
(*b*) A schematic representation of the essential elements of a rudimentary neutron diffractometer, upstream of the sample position (which would be located to the right of the beam exit slit of width *W* defined by a pair of opaque masks as indicated by the black rectangular blocks). A mosaic crystal (*e.g.* pyrolytic graphite) directs incident neutrons (by energy-selective Bragg reflection), originating from a temporally and spatially extended incoherent source, through a pair of slits, resulting in a quasi-monochromatic beam being incident on a sample. This beam is a collection of individual neutrons, each with an associated wave packet (in the drawing, Δ*r*_T_ indicates a measure of the packet transverse spatial width). The *j*th individual wave packet corresponds to one specific neutron that is a member of a collection of *N* similar neutrons. Each packet has a mean wavevector **k**_M_. (*a*) Another schematic illustrating how the beam is characterized by a distribution of packet mean wavevectors that define a geometrical angular divergence related to *W*/*L* = tan(Δθ_BEAM_). As is also shown, each packet is itself composed of a coherent distribution of basis states with corresponding eigenvector directions associated with a distribution characterized by a width Δθ_WP_ (where the subscript ‘WP’ indicates wave packet). The resultant picture is one in which both coherent and incoherent distributions of wavevectors corresponding to the individual neutron packets and beam, respectively, coexist. The monochromator and pair of apertures together define both the individual and collective properties of the packets and beam, respectively, as described in the main text.

A schematic representation of such a neutron beam is provided in Fig. 2 ▸(*a*), and Fig. 2 ▸(*b*) illustrates how such a beam might be realized in a typical reflectometer consisting of a mosaic monochromating crystal and a pair of slits. In practice, this monochromating crystal (*e.g.* pyrolytic graphite or PG) redirects incident neutrons (by energy-selective Bragg reflection) arriving from a temporally and spatially extended incoherent source on through a pair of slits, thereby creating a nearly monochromatic, collimated beam. The mosaic crystal is composed of perfect micro-crystal blocks which are randomly oriented about a mean according to a Gaussian distribution and which also possess an amount of translational disorder relative to one another. The collection of blocks therefore acts as a secondary incoherent, extended source wherein each individual block effectively produces directed, individually coherent, neutron packets. As will be discussed later, the monochromating crystal affects the transverse and longitudinal coherence of individual packets considerably.

(After emanating from the primary source and subsequent transport by a typical guide tube, neutrons incident on the monochromator may have already acquired wavefunctions with a similarly localized packet form owing to mirror reflection from the wavy surfaces of the guides – as discussed in Section 6.2.1. But even if a neutron packet incident on the monochromator were spatially extended enough to interact with more than one mosaic block at a time, the disordered spatial relationship between the blocks makes it less likely to result in a significant coherent interaction analogous to double- or multiple-slit interference. To our knowledge, no evidence for such interference exists.)

### 3.1. A general description of a wave packet

Consider next a more explicit description of a wave packet associated with one of the constituent neutrons in a beam. One mathematical representation of a free neutron wave packet that is widely adopted is a function Ψ(**r**, *t*) made up of a weighted distribution of plane waves which may be written as

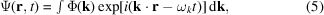

where **r** is the spatial coordinate, **k** is the basis-state wavevector, ω_*k*_ is the corresponding angular frequency, *t* is the time and Φ(**k**) describes the distribution of the basis states. This real-space representation and its momentum-space counterpart are, as is well known, related by a Fourier transform. The effective size and shape (in real space) of an individual neutron wave packet – including the extent over which a given wavefront has a uniform phase – is related to the coherent superposition of momentum basis states forming the packet through that Fourier transform. This connection can be expressed (famously) as an uncertainty relation which applies along each Cartesian direction, including the orthogonal directions perpendicular (transverse) and parallel (longitudinal) to the mean packet wavevector. The intrinsic uncertainties in position and momentum, corresponding to the widths of the distributions in momentum Δ*k* *_j_* and coordinate Δ*r* *_j_* for each rectangular component (*j* = *x*, *y*, *z*), are related. For the case of Gaussian wave packets (minimum uncertainty) this well known relation is

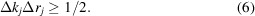

Although a single plane-wave basis state is a solution of both the time-dependent and time-independent Schrödinger equations of motion, in general the 3D wave packet of equation (5) satisfies only the explicitly time-dependent version.

Wave packets are shaped by the instrumental elements through which they travel, which modify the Fourier components of the packet. A wave packet, whether it be a photon or a massive particle, is characterized by a longitudinal coherence and a transverse coherence, parallel and perpendicular to the packet’s direction of motion, respectively. The longitudinal coherence depends on the length of the wave packet along its direction of propagation and the energy spread involved in its formation. This is determined primarily by the monochromator. However, it is the transverse component that is the principal subject of interest in the work reported here. The transverse coherence is defined as the width over which the wavefronts of the packet do not fall out of phase by more than a certain specified amount. In general, we will label this transverse coherence as Δ*r*_T_. As a specific example, for a wave packet composed of a superposition of plane waves possessing a Gaussian distribution of transverse wavevector components, the transverse coherence is related to the corresponding spatial distribution width. However, plane wavefronts that go through a narrow aperture, for instance, will take on a spherical form as a result of diffraction that can be described by the Huygens construction. In that idealized case, a simple definition of the transverse coherence can be derived as λ/Δθ (Kaganer *et al.*, 2001 ▸), where Δθ is the angular divergence imposed by the slit. In general, an aperture will reduce the transverse coherence, whereas diffraction from a crystal, as a result of the phase requirements of the Bragg condition, increases transverse coherence. Thus, an oriented microcrystalline monochromator, like highly oriented pyrolytic graphite (HOPG), can generate a neutron beam which has a certain degree of angular spread, but, by virtue of the diffraction of individual neutron wave packets, each neutron can possess a relatively large transverse coherence, as will be discussed in following sections.

In addition to the contributions that instrumental elements make to the preparation of a neutron state, however, the ultimate form of the wavefunction also depends on other fundamental quantum theoretical considerations pertaining to how the packet is initially created and subsequently evolves in space and time when associated with a freely propagating quantum particle. At present, the actual shape, size and composition of the packet embodied by Φ(**k**) for a neutron immediately upon emission from a temporally and spatially extended incoherent source (presumably formed in the process) or exactly how it changes thereafter is not definitively known. A considerable body of work on fundamental concepts in quantum theory exists regarding possible theoretical descriptions of spatially localized wavefunctions and the wave equations which govern them (*e.g.* Ghirardi *et al.*, 1986 ▸; Bassi *et al.*, 2013 ▸). This work introduces stochastic and nonlinear elements to a new dynamics that addresses the measurement problem and collapse of the wavefunction, which the Schrödinger equation does not, yet approaches the latter description in some limit. [Other discussions of possible neutron wave packet forms have been given (*e.g.* Utsuro & Ignatovich, 2010 ▸).]

Nonetheless, even if the exact form of the function describing the neutron packet were known, the solution of scattering problems involving wave packets in more than one dimension is significantly more complicated. Relatively few wave packet treatments of neutron scattering have been applied other than for theoretical work involving one-dimensional models (*e.g.* Berk, 2014 ▸, 2018 ▸; Dimeo, 2014 ▸). So, for practical reasons, it is necessary to adopt a simpler description of a packet wavefunction that preserves the spatial localization with wavefronts of limited transverse width and is also a reasonably good stationary-state solution of the time-independent Schrödinger equation of motion.

### 3.2. A simple model wave packet

To describe the effect that the neutron wave packet’s finite transverse spatial extent has on diffraction from material objects, a model can be adopted that is simpler than a full three-dimensional packet yet nonetheless preserves the essential consequences of a finite transverse spatial size. Short of describing the neutron wavefunction as a more realistic – but far more complicated – wave packet localized in all three dimensions, it is possible to obtain a wavefunction that is partially localized in the two orthogonal transverse directions (perpendicular to the mean propagation wavevector) but which is also a stationary-state solution of the time-independent Schröoedinger equation. (Explicit time dependence is not of direct significance since only elastic scattering from static systems is being considered, *i.e.* no time-dependent potentials or fluctuations are involved. Such a wavefunction can be constructed by a suitable superposition of wavevector directions about the *x* axis of propagation (*i.e.* a distribution of *y* and *z* components) such that the magnitudes *k* of all the component wavevectors are equal – that is, 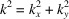 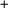 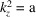 constant value. For appropriate distributions, say Gaussian, of *y* and *z* components of the wavevector (with the propagation direction along the *x* axis), the resultant waveform resembles a tubular-like wave localized in the **y** and **z** directions but extended along the *x* axis. This simplified waveform is similar (though not exactly identical) to the ‘quasi-monochromatic wave train’ solution of the approximate paraxial wave equation employed in elementary treatments of the diffraction of light (*e.g.* Hecht, 1998 ▸) and electrons. [A similar approximation has been adopted in the description of initial electron wavefunctions where orbital angular momentum is consecutively imparted by an appropriate device (Bliokh *et al.*, 2017 ▸).]

The form of this wavefunction in two dimensions (which suffices to illustrate the essential behavior of a packet in the material to follow) – one transverse along the *y* axis and the other longitudinal along the *x* axis – is given by

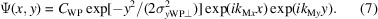

Here, the factor *C*_WP_ incorporates normalization constants associated with the wavefunction and the Gaussian distribution of the transverse components of the wavevector. σ_*y*WP⊥_ is the standard deviation of the width of the packet in real space and may be related to a measure of Δ*r*_T_ as defined in the previous section[Sec sec3.1]. *k*_M*x*_ and *k*_M*y*_ are the *x* and *y* components of the mean packet wavevector **k**_M_, respectively. (The inclusion of the *z* axis is straightforward and similar in nature to the treatment of the other transverse direction along **y**.)

In a typical example, an elongated packet waveform of this type for an individual neutron might have a distribution of plane-wave basis states corresponding to an angular range of wavevector orientations of the order of ɛ = 5′′ (2.424 × 10^−5^ rad) about the mean wavevector direction. In this case, the constraint that *k* ^2^ be a constant value (*e.g.* *k* = 2π/5 Å) results in a magnitude variation of the longitudinal wavevector component of approximately *k*(1 − cosɛ) = 2.94 × 10^−10^ *k*, whereas the transverse (perpendicular) component variation is *k*sinɛ = 2.424 × 10^−5^ *k* (or 8.25 × 10^4^ times larger). Another way of expressing the condition for the formation of such an elongated wave packet is to require that the magnitude of the width of the distribution of transverse wavevector components Δ*k*_⊥_ ≪ |**k**|, where **k** is along the longitudinal direction (the distribution of the magnitudes of the wavevectors in a ‘monochromatic’ neutron reflectometer beam, Δ*k*/*k*, is typically 0.01). (Moreover, since the extent of longitudinal or parallel localization of the packet, *i.e.* along the direction of propagation, depends upon the details of the interaction time with the moderator nucleus and any subsequent monochromation such as may be caused by crystal diffraction or a physical shutter device, the longitudinal packet dimension is not of particular import for elastic interactions. Therefore, an elongated packet function along the direction of propagation is acceptable.)

For present purposes, we will assume that neutron wavefunctions possessing nearly flat wavefronts of truncated lateral extent suffice to account for the essential effect of averaging over a finite in-plane area of a specularly reflecting material. That this is a reasonable assumption in practice is supported by the types of waveforms typically produced by slits and perfect micro-crystal blocks as examined in Section 4. It will also be assumed that the coherent superposition of the basis wavevectors composing an individual neutron wave packet is the same for all packets in the beam except that each packet has its own mean wavevector, indicating the direction of the neutron it describes.

## 4. Shaping a neutron wave packet within an instrument

Since the neutron optical elements of the instrument contribute to the formation of a neutron packet wavefunction in practice, it can be asked whether the type of simplified wavefunction introduced in equation (7) is a reasonable approximation. We therefore consider the form of the wavefunction that results from an interaction with a slit aperture and a micro-crystal block.

### 4.1. Single-slit aperture diffraction

Consider first the waveform generated by a single-slit aperture in two dimensions. We begin with an incident plane wave generating a scattered intensity as observed in the far field at a large distance *S* from the slit with *S* > *W* ^2^/λ, where *W* is the width of the aperture. In this Fraunhofer limit, the measured intensity at an angle θ, relative to the incident direction, is given by

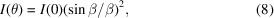

where β = (π*W*/λ)sinθ and θ is the angle from the bisecting perpendicular to the aperture at the center of the opening to a line connecting the slit center to a point on a detecting plane [*I*(0) represents the incident wave intensity; *e.g.* Hecht, 1998 ▸]. The square of the sinc function on the right-hand side of equation (8) has a central maximum with an FWHM Δθ_SSD_ (the subscript SSD indicates single-slit diffraction) approximately equal to the first zero of sincβ, which is given by θ = arcsin(λ/*W*).

**Figure 3:**
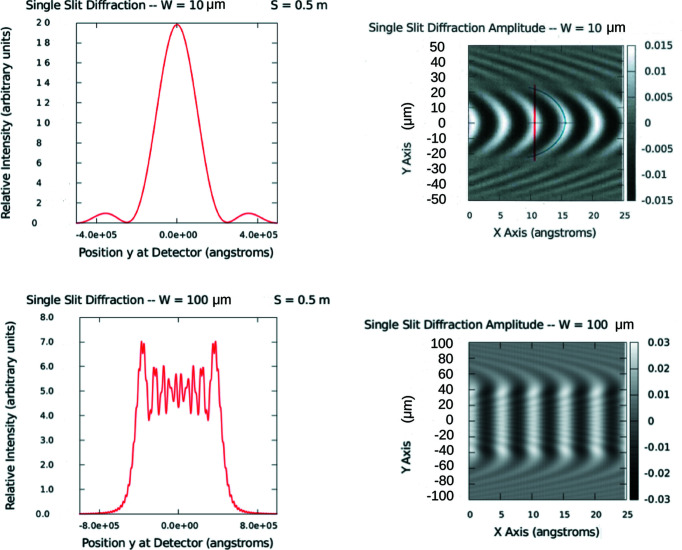
Intensity patterns (left-hand column) and the real parts of the diffraction amplitudes (right-hand column) from slit apertures corresponding to two of the examples listed in Table 1 ▸. The neutron wavelength is taken to be 5 Å. The intensity patterns are plotted at a point of observation a distance *S* (0.5 m) away from the aperture along the *y* axis, perpendicular to the direction of propagation of the wave incident on the slit. For the amplitude plots, the *y* axis is also perpendicular to the incident beam direction, whereas the *x* axis extends a distance of five neutron wavelengths along the direction of propagation back from the farthest observation point at a distance *S* (0.5 m). One measure of the distance Δ*r*_T_ over which a given wavefront is uniform in phase to within one wavelength can be determined by examining two consecutive wavefronts propagating along the *x* axis, as pictured in the upper right-hand plot. Choose an arbitrary leading wavefront. Draw a curve (shown as blue) along that wavefront’s ridge of relative maximum amplitude. Next, construct a straight line (shown as red) parallel to the transverse *y* axis through the central maximum (at *y* = 0.0) of the wavefront immediately following (to the left). The *x* coordinates of either of the intersections of the blue curve and red line differ from that of the central maximum of the leading wavefront by one wavelength. For the 10 µm slit, this measure corresponds roughly to that obtained from the first minimum of the intensity on either side of the central maximum. The 10 µm-wide slit produces a pattern at 0.5 m that is in the far-field limit, while that of the 100 µm aperture is well within the near-field region.

However, it is of more practical interest to be able to determine the single-slit diffraction pattern over a broader range of distances and aperture widths. Neglecting higher-order multiple scattering effects, a simple Huygens–Fresnel wavelet construction can provide a relatively accurate picture of both the amplitude and intensity distribution of the waveform emanating from this single aperture over a significant range of distances from the near-field Fresnel region out to the far-field Fraunhofer limit. The results of such a Huygens–Fresnel construction for a variety of pertinent aperture widths and distances to a plane of observation are represented in Fig. 3 ▸ and Table 1 ▸. [The diffraction of neutrons by slit widths of the order of 100 µm was first conclusively demonstrated by Zeilinger *et al.* (1988 ▸).] Considering the amplitude waveforms on the right of the figure it is clear that the widths of the wavefronts propagating outward from the aperture possess a finite lateral extent over which the probability amplitude is of significant magnitude and of uniform phase within one wavelength. One measure of the lateral distance over which a given wavefront is uniform in phase to within one wavelength can be determined by examining two consecutive wavefronts propagating along the *x* axis as described in the caption for Fig. 3 ▸.

Note that the Huygens construction of the wave diffracted through the narrower 10 µm slit shown at the top of Fig. 3 ▸ produces the curved surface of a Gauss–Laguerre wave packet. On the other hand, for the 100 µm aperture, the wavefront is more planar over a certain width. This difference corresponds to being in the Fraunhofer as opposed to the Fresnel zone. Consequently, the criteria for defining the transverse coherent extent Δ*r*_T_ of the wavefront can be different to some degree for the two cases.

Thus, even if the neutron wavefunction approaching the slit is represented by a single plane wave, the interaction with any aperture of finite size transforms the wavefunction into one that is localized in space to a finite extent in a transverse direction. And although the transverse width continues to increase with distance from the aperture, as described by the Fraunhofer diffraction formula, it remains of finite size at a finite distance away from the aperture.

Unless the incident wavefront spans the width of a given aperture, the standard single-slit diffraction pattern will not be formed, although in some cases diffraction from one of the edges of the masks defining the aperture may occur. But in general, if the wavefront has a transverse extent less than the width of the aperture, the aperture acts primarily in the geometrical optics limit to define the spatial and/or angular range through which the mean wavevectors of individual neutron packets emanating from an upstream source (be it a point or extended) can pass. A pair of slits in series can therefore also define the angular divergence of a beam of individual neutrons, each with its own corresponding packet, as will be discussed further in Section 5.

### 4.2. Diffraction by a perfect micro-crystal mosaic block

The other basic neutron optical element of the reflectometer consists of a perfect micro-crystal block. The monochromator device as it is drawn in Fig. 2 ▸ is intended to represent a mosaic crystal composed of an angular distribution of perfect single-crystalline blocks of finite dimensions (*e.g.* pyrolytic graphite) which selects and redirects [via Bragg diffraction from the (002) atomic planes] a fraction of the neutrons incident upon it through the pair of downstream apertures. Consider the limiting case where the transverse spatial extent of an individual neutron wave packet that is incident on such a monochromator is sufficiently small that it can only interact with one perfect single-crystal mosaic block at a time. [The mosaic blocks composing the monochromator are essentially independent of one another because of their random orientations, translations and sizes (this applies to both horizontal and vertical directions in the nominal reflecting plane of the monochromator). Although a relatively small degree of order may exist between two or more individual blocks, resulting in constructive interference if they are illuminated simultaneously by the same incident wavefront, this is not observed to be a predominant effect in practice. To a good approximation, each block scatters coherently, whereas the collection acts as an incoherent source.]

Each mosaic block (with dimensions typically of the order of several micrometres; Gerlach *et al.*, 2015 ▸) can then be treated as a miniature monochromating device which – by the coherent Bragg reflection process – helps to define the transverse and longitudinal distributions of wavevector components corresponding to the set of basis wavefunctions that compose an individual neutron wave packet. In this case, the mosaic blocks, which are not necessarily all of exactly the same size or uniformly spaced from one another, are randomly oriented according to a Gaussian distribution of angles about a mean normal direction. Although each separate block is a coherent reflector of single neutrons, collectively the ensemble of blocks can be considered to be a spatially incoherent secondary source of a beam of neutrons. This distribution of mosaic blocks is analogous to a spatially incoherent distribution of apertures. But in contrast to the spherical waves that emanate from point sources isotropically, the blocks radiate over a limited range of preferred directions and with a quasi-monochromatic bandwidth.

The finite in-plane dimensions of the stack of reflecting atomic planes affects the distribution of basis-state functions composing the reflected neutron wave packet and thereby the area over which a component wavefront of the packet is of uniform phase. As will be shown below, at sufficiently large distances, in the far-field or Fraunhofer limit, and under appropriate initial conditions, the packet wavefronts generated by a mosaic block are comparable to those of truncated plane waves or the elongated monochromatic wave trains discussed in Section 3.2.

**Figure 4:**
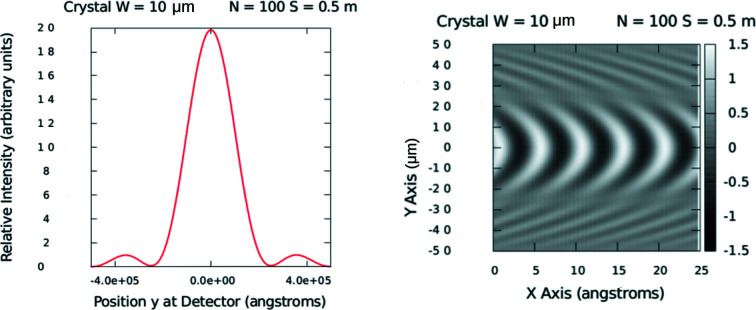
Results of a Huygens–Fresnel calculation assuming a generic crystal 10 µm wide with 100 reflecting atomic planes spaced 5 Å apart from each other, and with 1000 atomic source points per plane. The wavelength of the radiation was taken to be 5 Å as well, and the distance between crystal face and point of observation of the reflected wave was 0.5 m. This two-dimensional block of source points was taken to be illuminated by plane waves in phase as though the Bragg diffraction condition was effectively satisfied. As in the case of an aperture of the same width, the reflected wave has a well defined lateral dimension which at 0.5 m from its source has a uniform wavefront (to within one wavelength) over a lateral extent of approximately 22 µm – similar to that produced by the single aperture of the same width. On the left is a plot of intensity versus position on a perpendicular detection plane a distance 0.5 m away from the crystal. The horizontal axis of the wave amplitude plot on the right is along the mean direction of propagation, covering a distance of approximately five wavelengths up to the detection plane at 0.5 m at the right terminus. The vertical axis is along a perpendicular direction, and the degree of shading indicates the relative amplitude of the reflected wave.

A semi-quantitative picture of the scattering produced by a single-crystalline mosaic block of given dimensions can be obtained by performing the same type of Huygens–Fressnel wavelet construction as was applied to the aperture in Section 4.1. Wavelets are taken to emanate from each atomic source point isotropically (multiple scattering processes are neglected). For this simple model calculation, the single two-dimensional block of atomic source points was taken to be illuminated by plane waves in phase such that the Bragg diffraction condition was effectively satisfied. Shown in Fig. 4 ▸ is the result of such a calculation assuming a generic crystal, 10 µm wide with 100 reflecting atomic planes (lines in two dimensions) spaced 5 Å apart from each other, and with 1000 atomic source points per plane (or line in two dimensions). A single wavelength of 5 Å was assumed, and the distance between crystal face and point of observation of the reflected wave was 0.5 m. The reflected wave has a well defined lateral dimension, which at 0.5 m from its source has a uniform wavefront (to within one wavelength) over a lateral extent of approximately 22 µm – similar to that produced by a single aperture of the same width. So it is expected that for typical PG monochromators, the reflected neutrons have wavefunctions with transverse dimensions of the order of tens of micrometres at a distance of a metre or so away (this is consistent with the diffraction measurements described below and elsewhere; *e.g.* Treimer *et al.*, 2006 ▸).

The reflected neutron wave amplitude from a given block is a result of the constructive interference that occurs because of the periodic structure of parallel atomic planes in that block. Thus, the number of contributing reflecting planes can also affect the transverse width of the wavefronts of the reflected neutron packets. This effect can be examined by applying the traditional methods employed to analyze the diffraction from perfect and mosaic crystals where primary and secondary extinction of an incident wave play a significant role. Such considerations are beyond the scope of this article (further discussion can be found elsewhere; *e.g.* Majkrzak *et al.*, 2019 ▸; Zachariasen, 1945 ▸).

Given the results of the calculations described above, a reasonable practical approximation of a neutron wave packet can be made on the basis of the 2D wave train function introduced earlier, in which the wavevector magnitudes for all plane-wave components are nearly the same, with wavefronts that are nearly planar truncated sheets. The dimensions of these finite-sized wavefronts can be empirically determined by the two methods to be described in Section 6. Once an estimate of the extent of the wavefront is made, analysis of specular reflection data can be performed accordingly by taking the finite transverse extent of the wavefront into account.

## 5. Defining the beam angular divergence by a collimating pair of slit apertures in series

The combination of a collimating pair of slit apertures and a perfect crystal mosaic block can be regarded as the basic components typically employed to prepare an incident beam on a neutron reflectometer at a steady-state source.

In addition to the packet-shaping action of a slit via diffraction, a pair of apertures in series can act in unison to define the distribution of wave-packet directions within a beam. If the aperture widths are sufficiently large that diffraction effects are negligible, then the primary effect of the apertures is to define a collective beam contribution to angular divergence with an approximate FWHM α, where tanα = *W*/*L*, *W* is the slit width and *L* is the distance between the pair.

As an example, for *W* = 1 mm, the diffraction width predicted by the far-field Fraunhofer expression of equation (8) for λ = 5 Å is Δθ_SSD_ = 5.0 × 10^−7^ rad (2.865 × 10^−5^°), whereas a 0.1 mm slit width would give 5.0 × 10^−6^ rad (2.865 × 10^−4^°). On the other hand, the geometrical angular widths Δα = arctan(*W*/*L*) as defined by a pair of apertures of the same width *W* a typical distance *L* = 1500 mm apart are 6.67 × 10^−4^ rad (3.82 × 10^−2^°) and 6.67 × 10^−5^ rad (3.82 × 10^−3^°) for *W* = 1 and 0.1 mm, respectively. Thus the fractional increase in angular divergence caused by diffraction over that due to the geometrical width is only approximately 0.075 and 7.5%, respectively.

Note that there is no unique combination of *W* and *L* that defines a particular geometrical angular divergence Δα. In the absence of appreciable diffraction broadening from the slits, this geometrical angular divergence is constant with increasing distance downstream at any point of observation. It can only be meaningfully associated with the beam divergence arising from a distribution of mean wavevectors of the individual neutron packets composing a beam. Given the physical description of the instrumental components that define the beam and individual neutron wavefunctions presented above, an accurate representation of the beam profile for an actual instrument can be calculated.

According to the Huygens–Fresnel numerical calculation discussed in the previous section, diffraction by a slit of width 0.025 mm for 5 Å-wavelength neutrons produces a wave train with a transverse dimension that is roughly the same as the slit width at a distance of 1 m away, assuming of course that the wavefront of the incident packet was of sufficient planarity and width to span the aperture in the first place. The diffraction broadening by such a slit is only several arcseconds (∼3.44′′). This is negligible compared with the angular divergence (more than 1°) defined by the width of the monochromator source (approximately 25 mm) that illuminates the first downstream slit roughly a metre away. Thus, we can, to a good approximation, view the first (upstream) slit of the collimating pair of slits in series as a uniform (in space and angle) source which subsequently illuminates the second (downstream) slit.

However, given the widths and distance between the slits for the actual instrumental configuration used in the phase-grating measurements to be described in Section 6 (and in Appendix *A* [App appa]), the geometrical angular divergence of a beam defined by and emanating from the pair is 2.89 × 10^−5^ rad (1.66 × 10^−3^° = 0.0995′ = 5.97′′). Thus the beam defined by this pair of slits together has a geometrical angular divergence that is comparable (roughly twice as large) to the diffraction broadening associated with the downstream slit.

**Figure 5:**
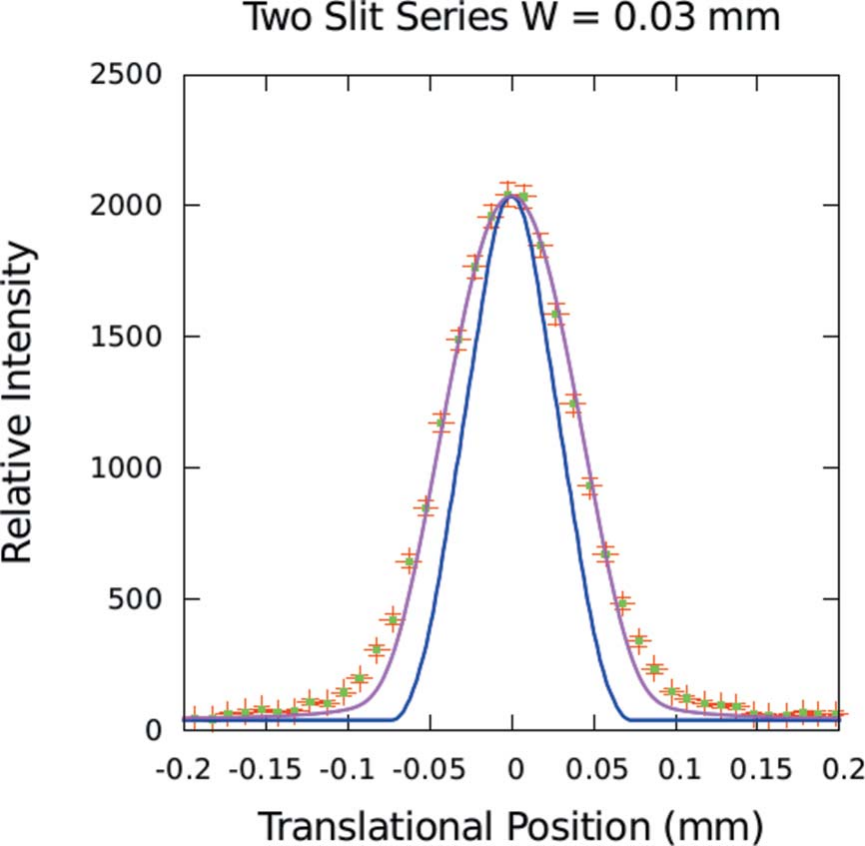
Numerical calculation (purple curve) of the beam profile expected to be projected onto the detector line on an instrument as compared with an actual measurement (points with error bars which represent ± one standard deviation and a corresponding confidence interval of approximately 68%). The computed curve is not a fit but only scaled to the measured intensity. This calculation takes into account the affect of the geometrical angular divergence (defined by the pair of slits) on a summation of diffracted intensity patterns from the second slit downstream – as predicted by the standard Fraunhofer diffraction formula for multiple source points across the width of the first (upstream) slit. Another calculation, corresponding to what would be expected based on a consideration of geometrical ray optics alone (blue curve), is also plotted. The agreement in the case in which both geometrical and diffraction effects are included is markedly better.

For the specific values described immediately above (and in Appendix *A* [App appa]), a numerical calculation of the beam profile expected to be projected onto the detector line of the instrument can be performed and compared with an actual measurement. The results are shown in Fig. 5 ▸. In the calculation, the geometrical angular limits defined by the pair of slits were taken into account and intensities – as given by the standard Fraunhofer diffraction formula – from source points across the width of the first (upstream) slit were summed.

In Fig. 5 ▸, the measured data are compared with the results of two calculations, one that included both geometrical and diffraction effects and another corresponding to what would be expected for geometrical ray optics alone. The computed curves are not fits but only scaled to the measured intensity. A slightly larger slit width of 0.030 mm was found to be in better agreement than the nominal value of 0.025 mm of the shim stock spacer used to define the gap. This is likely to be due to non-perfect alignment of the slits with respect to the vertical axis and to imperfections in the machined edges of the 1 mm-thick Cd masks, and possibly also due in part to mirror reflection and refraction as well as diffraction from the mask edges by packet wavefronts of insufficient lateral extent to span both mask edges simultaneously.

To summarize, the width of the distribution of transverse wavevector components of the basis states constituting an individual neutron packet is related to the spatial transverse width of the packet through the uncertainty principle of equation (6). The angular divergence associated with the collection of packets composing a beam, on the other hand, is related to the width of the distribution of the corresponding mean packet wavevector directions as defined by geometrical constraints, *e.g.* by a pair of slits in series.

## 6. Measurement of coherent wavefront width

From the preceding discussion, it follows that it is natural to distinguish between two different characteristics of a neutron beam – one pertaining to a collective angular beam resolution and the other to the transverse extent of each individual constituent neutron packet’s wavefronts. But to do so in practice can be problematic since both the collective aspects of the beam and the individual properties of each constituent packet are defined to some degree by the very same instrumental devices such as slit apertures and single-crystal monochromators.

Nonetheless, two ways to determine the effective transverse spatial extent of the neutron packet are described here. One method involves diffraction from gratings for which the correlation length of the periodic structure has been independently determined. In the other method, originally suggested in earlier work (Majkrzak *et al.*, 2014 ▸), the transverse extent of a wavefront can be experimentally determined through specular reflection measurements at glancing angles from patterned thin films of known composition and structure. New experimental data and analyses are presented here which more precisely quantify both the advantages and limitations of this method. Although this glancing-angle reflection method is found to be largely independent of the distribution of packet mean wavevector directions of individual neutrons in a beam (geometrical angular divergence), it is susceptible to waviness or deviations from perfect flatness of the surface of the substrate (figure error) on which the film structure is deposited, as will be discussed below.

### 6.1. Diffraction from phase gratings in near-normal transmission

Consider a beam made up of neutrons with the 2D longitudinally elongated packet wavefunctions described above, illuminating a phase grating at normal incidence (in transmission geometry). For neutrons with a 5 Å nominal wavelength, single-crystal silicon has a scattering length density (2.1 × 10^−6^ Å^−2^) that produces a π phase shift over a distance of about 30 µm. By etching a parallel set of grooves of this depth with a uniform width and spacing and of rectangular cross section into a plate of perfect single-crystal silicon, a transmission phase grating can be fabricated. The number of grating periods that simultaneously interact (*i.e.* coherently) with an individual incident neutron depends on the transverse extent of a neutron packet wavefront over which the phase is sufficiently uniform. For example, if the wavefront uniformly spans a width on a π-phase-shift grating equivalent to *N* periods, the diffracted intensity *I*_PG_ in the far-field or Fraunhofer regime depends upon *N* directly as given by

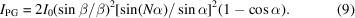

**Figure 6:**
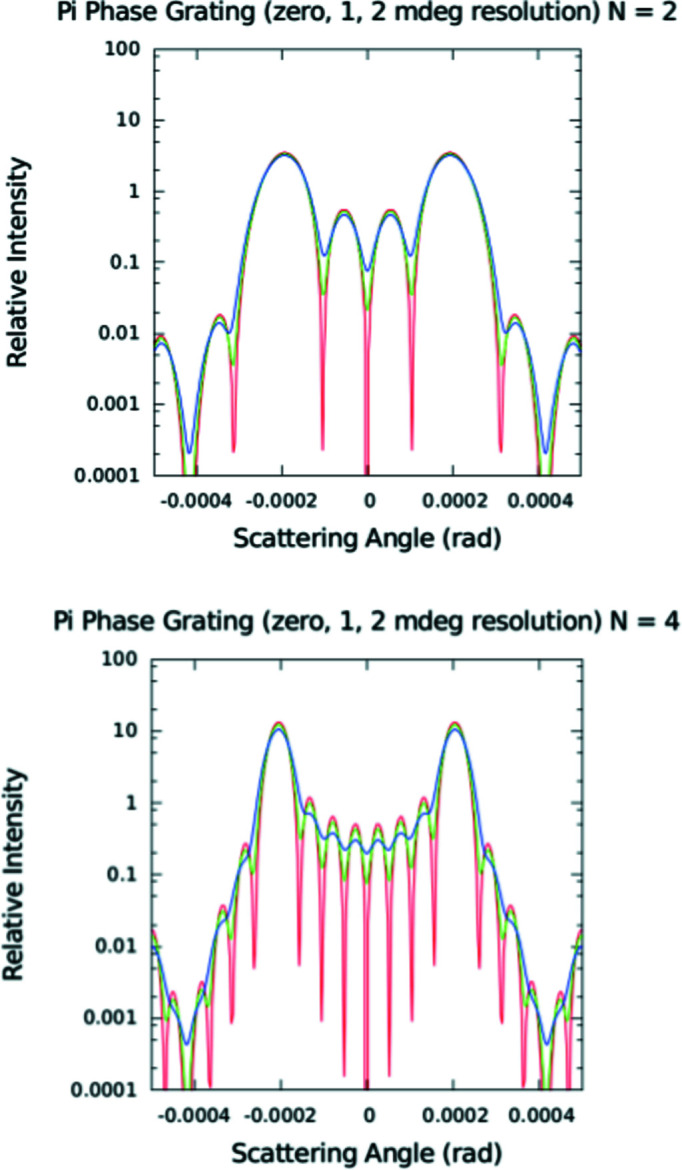
Diffraction patterns calculated for a model π-phase-shift grating with equal column and trough thicknesses at a 5 Å neutron wavelength. One set of patterns corresponds to coherent contributions from two grating periods (top) and the other set to contributions from four (bottom). The period of the grating is 2.4 µm. Assuming that the grating itself is perfectly uniform, the number of coherently contributing periods then depends on the transverse width of the neutron wavefront over which the phase is of the requisite uniformity. The geometrical angular divergence of the incident neutron beam – which corresponds to a distribution of transverse components of packet mean wavevectors – determines how well the features of the pattern are resolved. Both figures include cases for zero-beam angular divergence (red lines) and two other finite values (3.6 and 7.2′′, green and blue lines, respectively) convoluted with the natural pattern. Note that the general shape of the pattern is preserved, while the widths and magnitudes of the principle and subsidiary reflections are affected by the degree of the geometrical angular divergence of the incident beam. This description in which a distinction can be made between the effect of the intrinsic transverse width of an individual neutron packet and that of the beam geometrical angular divergence is supported by measurements reported here (which are to follow) and those of others (Treimer *et al.*, 2006 ▸).

Here *I*_0_ is the incident intensity, and α = (*ka*/2)sinθ and β = (*kb*/2)sinθ, in which *b* is the groove width, *a* is the grating spacing or period, and *k* = 2π/λ (*k* and λ are the nominal neutron wavevector and wavelength, respectively). The angle θ is defined similarly to the angle of diffraction for the case of a single slit. Note that equation (9) is explicitly for the case where the neutron wavevector is exactly perpendicular to the plane of the grating. For a beam composed of packets with a distribution of mean wavevector directions, *i.e.* with a geometrical angular divergence, the intensity contributions, properly weighted according to the particular distribution (*e.g.* Gaussian), must be summed over the range of incident angles in that distribution. For relatively narrow angular distributions, the center of the diffraction pattern corresponding to a given incident angle is, in the small-angle approximation, effectively shifted from that for normal incidence by an amount approximately equal to the difference in the given angle of incidence from the normal, thereby causing a smearing of the observed diffraction pattern. Fig. 6 ▸ shows diffraction patterns calculated for a model π-phase-shift grating with equal column and trough widths at a 5 Å neutron wavelength. (Although not shown in Fig. 6 ▸, a plot of *I*_PG_ versus θ for *N* = 1 reveals that the positions of the principal maxima on either side of the origin are markedly shifted compared with those for *N* > 1.) Table 2 ▸ compares the width of the transverse wavevector component distribution Δ*k*_TWP_ associated with an individual wave packet – possessing a corresponding spatial transverse width Δ*r*_TWP_ – with that of the packet mean wavevectors Δ*k*_MT(BEAM)_ contained within the beam [hereafter the subscript (BEAM) will be abbreviated to (B), as, for example, in Δ*k*_BMT_].

**Figure 7:**
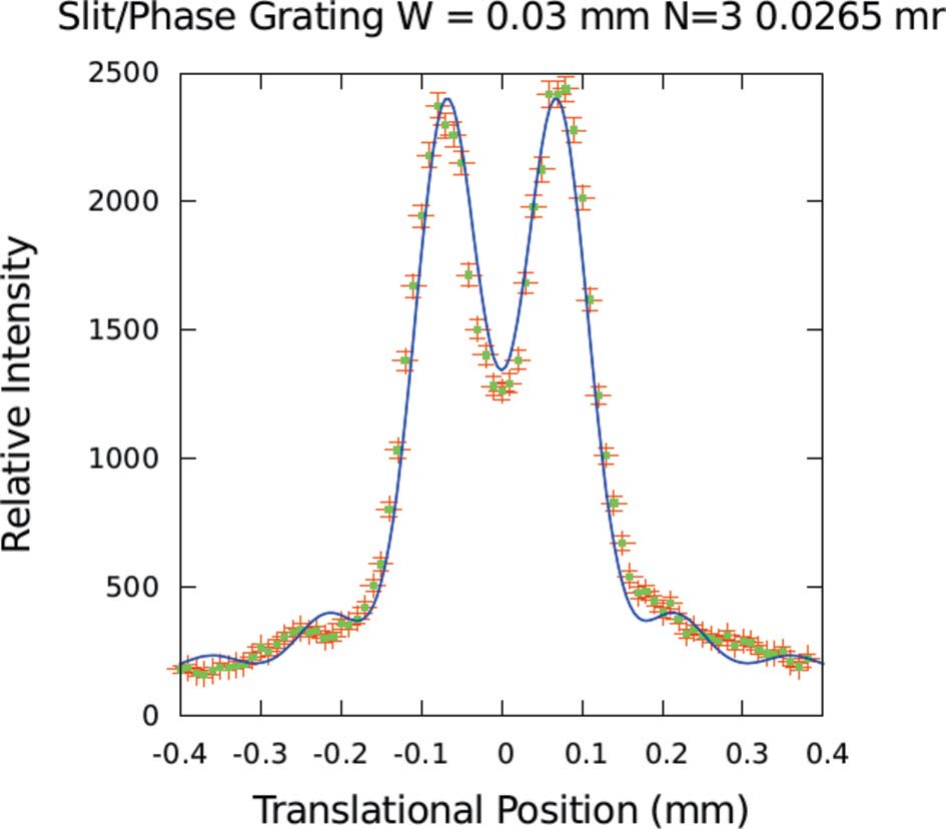
Diffraction pattern measured (symbols with error bars which represent ± one standard deviation and a corresponding confidence interval of approximately 68%) from an 8 µm-period π-phase-shift grating with equal-thickness rectangular troughs and columns etched in single-crystal silicon at a neutron nominal wavelength of 5 Å. Also plotted in this figure is a calculated diffraction pattern based on the phase-grating formula of equation (9) and assuming an incident illuminating beam exactly as described in the preceding section for the pair of slits which resulted in the profile of Fig. 5 ▸ (the phase grating was located 495 mm away from the second downstream slit) – that is, both geometrical and diffraction effects in forming the beam of wave packets incident on the grating by the pair of slits were taken into account. In the computed phase-grating diffraction pattern, both the distribution of geometrical angles in the incident beam and the transverse dimension of an individual neutron packet wavefront were included. The model calculation was not a fit to the data but only scaled to the measured intensity. The best agreement between the data and model was obtained for *N* = 3 and for a slight curvature of the grating substrate amounting to about 2.65 × 10^−5^ rad (5.47′′). (This bending might alternatively be attributed to a curvature of a neutron packet wavefront – which was originally taken to be perfectly flat but limited to a 24 µm finite lateral extent.) The general agreement between measurement and model calculation is good, although details in the wings are not resolved. This is likely to be due to relatively small effects involving mirror reflection, refraction and diffraction from the mask edges of the slits defining the incident beam.

Fig. 7 ▸ shows a diffraction pattern measured on the MAGIK (formerly AND/R; Dura *et al.*, 2006 ▸) reflectometer at the NCNR from an 8 µm-period π-phase-shift grating with equal-thickness rectangular troughs and columns etched in single-crystal silicon (Lee *et al.*, 2009 ▸) at a nominal neutron wavelength of 5 Å. The MAGIK reflectometer was configured for typical reflection measurements except for the special modifications described in Appendix *A* [App appa]. However, the grating diffraction measurements were performed in transmission with the incident beam perpendicular to the nominal plane of the grating (which was located at the sample position). Also plotted in this figure is a calculated model diffraction pattern based on the phase-grating formula of equation (9) for comparison. The best agreement between the data and model was obtained for a pattern with *N* = 3 – which is markedly different from that for a pattern with *N* equal to either 2 or 4 (as can be seen by comparing Figs. 6 ▸ and 7 ▸). The fit to *N* = 3 indicates that the transverse coherent extent of an incident neutron wave packet is approximately 24 µm for this instrumental configuration. (We also considered other potential contributions, including diffraction and mirror reflection from a single edge, but concluded that they would produce relatively small effects. We believed that we could not adequately distinguish between such separate contributions at this level and, therefore, did not take them into account in the final analysis to avoid the possibility of over-interpreting the data.)

In related work by Treimer *et al.* (2006 ▸), diffraction patterns were measured for single slits of various widths (including 100 µm) as well as for multiple-groove gratings (periods of 16 and 32 µm) with neutrons prepared on an ultra-small-angle neutron scattering (USANS) instrument [nominal wavelength of 5.248 Å using an HOPG(002) pre-monochromator and a pair of seven-bounce channel-cut Si(111) crystals as monochromator and analyzer]. The definition of the beam angular divergence by channel-cut Si crystals instead of a simple pair of slits results in a significantly cleaner beam, free from spurious artifacts potentially caused by edge effects of the masks used in our instrumental setup discussed above. In Treimer *et al.*’s grating diffraction experiments, measurements were performed at two different values of the incident beam angular divergence – 1.4 and 5.7′′ FWHM. The diffraction pattern features were found to be better resolved at the tighter angular resolution of the beam, as would be expected from the description of a beam of neutrons that we have presented above. Moreover, it was observed that the diffraction patterns obtained in their experiments could be fitted to a high degree of accuracy assuming a transverse extent of the neutron packet wavefront of 80 µm FWHM, irrespective of whether the angular spread of the beam was 1.4′′ (2.33 × 10^−2^′ = 3.89 × 10^−4^° = 6.79 × 10^−6^ rad) or about four times larger at 5.7′′ (2.76 × 10^−5^ rad). If the distribution in the transverse wavevector components of an individual neutron wave packet were to be attributed to these beam geometrical angular divergences Δα, a transverse wavevector distribution subsequently computed from Δ*k*_T_ = *k*Δα would predict transverse wave packet widths only of the order of Δ*r*_T_ = 6.15 and 1.51 µm, respectively, via the uncertainty relation Δ*k*_T_Δ*r*_T_ = 1/2 [equation (6)]. This would clearly be at odds with the experimental finding of 80 µm for the transverse extent of the neutron packet wavefront. The evidence is consistent with there being two distinct distributions – one being that of the mean wavevector directions of all of the neutron packets composing the incident beam and the other a distribution of transverse wavevector components associated with the coherent superposition of momentum basis states that form a single neutron packet wavefunction.

The difference between the transverse coherent extent of the packet determined by our experiment and that in the work of Treimer *et al.* (2006 ▸) is presumably due to the details of the manner in which the incident neutron packets were prepared in the respective instruments on which the measurements were performed.

### 6.2. Specular reflection from a patterned film structure at glancing angles of incidence

Normally, in scattering studies of ordered systems, knowing the resolution of the instrument establishes limits on the scale over which the correlations between structural features in a given material can be assessed. Conversely, a known correlation in a periodic sample can be used as a measuring tool to infer the transverse coherence extent of a neutron packet wavefront as described, for instance, in Section 6.1. However, there is another type of measurement that can be sensitive to the extent over which the phase of a wavefront is uniform (Majkrzak *et al.*, 2014 ▸). Through specular reflection at glancing angles of incidence, the structure and composition of a material object such as a patterned thin film on a flat surface can be tailored to serve as a probe of an individual wave packet’s transverse spatial extent.

**Figure 8:**
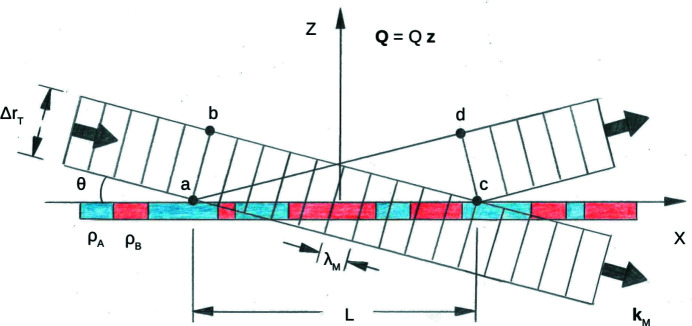
Schematic representation of a wave packet of rectangular form interacting with a planar sample of inhomogeneous SLD in plane (two values, ρ_A_ and ρ_B_). This picture illustrates how, for elastic, coherent, specular scattering, the area of the scattering surface that a wavefront of constant phase interacts with along the horizontal axis in the figure is actually its transverse dimension Δ*r*_T_ projected a length *L* across the surface. The other, orthogonal width (along an axis perpendicular to the plane of the figure itself) in the plane seen by the wavefront is not amplified but equal to whatever the packet width is in that direction. The lower edge of the *j*th wavefront intersects the sample surface first, on the left, and then the upper edge a distance *L* further along. Note that the distances *a* to *d* and *b* to *c* are equal – applying the Huygens–Fresnel wavelet construction shows that in the specular condition the incident planar wavefront *ab* is exactly in phase with the reflected wavefront *cd* (assuming a perfectly flat material reflecting surface).

As already discussed, the spatial extent over which the wavefront is sufficiently uniform is a fundamental quantity in determining the nature and degree of the coherent scattering that is possible from a given object. Fig. 8 ▸ schematically represents a wave packet similar in form to the elongated wave train described earlier but of rectangular cross section (conceptually useful as a simple image of successive wavefronts of constant phase), interacting with a planar sample of inhomogeneous SLD (two values, ρ_A_ and ρ_B_). This image shows that, for elastic coherent specular scattering, the distance on the scattering surface that a wavefront of constant phase successively interacts with (*i.e.* along the horizontal axis in Fig. 8 ▸) is proportional to its transverse dimension Δ*r*_T_ projected onto a length *L* across the surface. For a mean wavevector **k**_I_ making a glancing angle θ,

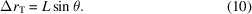

The other, orthogonal, width of the wavefront (along an axis perpendicular to the plane of the figure itself) intersecting the scattering surface is not geometrically enhanced but is equal to whatever the packet width is in that direction.

Recalling equation (2) and earlier discussion, the effective SLD for the area of the sample seen by the packet with a transverse dimension Δ*r*_T_ is the average density within the projected in-plane area, *i.e.* a weighted area average of ρ_A_ and ρ_B_ (pictorially, some combination of blue and red ⇒ some shade of purple). Note that this average SLD pertains only to any resultant specular scattering [*i.e.* where the wavevector (and momentum) transfer is strictly perpendicular to the mean surface normal]. (Non-specular scattering can also occur at other angles but is not of relevance to the present discussion.) Conversely, if the projected length *L* were sufficiently less than the dimensions of the areas corresponding to a single scattering length density, either ρ_A_ or ρ_B_, then the specular scattering would be observed to be a weighted incoherent sum of two independent reflected intensities, each associated with one or the other separate homogeneous region of SLD.

It has been previously demonstrated that patterned thin films of known structure (*e.g.* stripes) can be used to infer Δ*r*_T_ for neutron wave packets (Majkrzak *et al.*, 2014 ▸, and references therein). Referring again to Fig. 8 ▸, suppose that the materials of two different SLDs, ρ_A_ and ρ_B_, are rearranged to be of uniform (and equal) width and spacing along the horizontal *x* axis to form an alternating periodic grating structure (with continuous bars of material along the *y* axis perpendicular to the plane of the figure). For the analysis to follow involving specular reflection, the structure’s periodicity is not essential. However, selection of a set of samples with regular patterns of various periods does facilitate experimental control of the sizes of the in-plane areas of different SLD which are covered by the projection of an incident wavefront. For elastic specular reflection at glancing angles of incidence (typically a fraction of a degree for neutrons of 5 Å wavelength), the wavevector transfer *Q* = 2*k*_M_sinθ_M_ = *Q_z_* is along the *z* axis, perpendicular to the *xy* plane of the surface. The mean wavevector of a neutron packet, **k**_M_, has an incident (I) and final (F) direction, prior to and after scattering, respectively, whereas the magnitudes of the two wavevectors are equal since the scattering is elastic.

**Figure 9:**
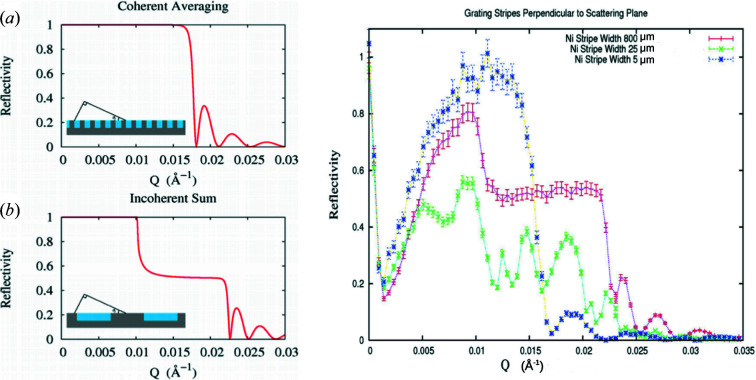
Summary of one of the principal results of previous work (Majkrzak *et al.*, 2014 ▸) in which it was originally demonstrated that the specular reflectivity about the critical edge for external mirror reflection is a sensitive measure of the projected length of a neutron wavefront [after Figs. 7 ▸ and 15 ▸ of Majkrzak *et al.* (2014 ▸); reprinted with permission; copyright (2014) American Physical Society.] (The quantity along the horizontal axes is *Q* = *Q_z_*, corresponding to the specular condition.) (*a*) Model specular reflectivity curve corresponding to an effective coherent averaging of two different SLDs. In the real-space schematic of the patterned thin-film structure in the inset, the material for the periodic rectangular film structure is the same as that of the substrate and is taken to have the SLD of Si; the troughs in between, on the other hand, are filled with material having the SLD of ordinary Ni (bar and trough widths are equal). Only a single critical *Q* is observed. (*b*) Model specular reflectivity curve corresponding to an incoherent sum of two independently scattering areas of in-plane SLD in the film. Two distinct critical *Q* values appear in this case. Both of the model reflectivity curves plotted in (*a*) and (*b*) were calculated for the case of perfect instrumental resolution – *i.e.* a monochromatic beam with no angular divergence. On the right-hand side of the figure are shown experimental specular reflectivity data for the two limiting cases (in addition to an intermediate case) (Majkrzak *et al.*, 2014 ▸).

Now consider how the position of the critical edge at the angle for total external reflection can be used as an indicator of the projected coherent extent of neutron packet wavefronts. As represented schematically in Fig. 9 ▸, if the projected wavefront is of sufficient extent to effectively average over the two SLD values, one associated with the grating bars and the other with the troughs, then the specular reflection corresponds to a coherent scattering process for a material with a uniform SLD that is the average of that of the bar and trough. In this case a single critical edge will be observed. If, on the other hand, the widths of the bar and trough are each sufficiently larger than the neutron wavefront’s projected dimension, then the observed specular reflectivity will represent the area-weighted incoherent sum of the reflected intensities for the bar and trough separately. In these circumstances, two distinct critical edges are manifest. Also shown in Fig. 9 ▸ are the principal experimental results summarizing earlier work (Majkrzak *et al.*, 2014 ▸). Relevant critical *Q*_c_ values are given in Table 3 ▸.

Nonetheless, an ensemble of similarly shaped packets composing the beam can have different mean wavevector directions relative to an average value, as characterized by the angular range Δθ_BM_ (which can be defined as the FWHM of such a distribution). As originally depicted in Fig. 2 ▸ as Δθ_BEAM_, this instrumental beam angular divergence Δθ_BM_ is distinct from Δθ_WP_ which corresponds to the intrinsic transverse wavevector uncertainty Δ*k*_TWP_ associated with an individual packet. The instrumental beam angular divergence corresponds to a directional distribution of *N* mean packet wavevectors **k**_M_, each of which has a one-to-one correspondence to a specific one of the *N* individual neutrons and their associated state or packet wavefunctions within the ensemble composing the beam. Although there exist two angular distibutions, Δθ_BM_ and Δθ_WP_, only Δθ_WP_ is directly connected with the transverse coherent extent of a neutron packet wavefront Δ*r*_T_ through the uncertainty relation Δ*k*_TWP_Δ*r*_T_ ≥ 1/2. So how then might the geometrical angular divergence of a beam affect the determination of the transverse wavefront width through the analysis of specular reflection in the vicinity of the critical angle as discussed above?

Let us first briefly review what the conventional measure of the instrumental resolution – in terms of wavevector transfer **Q** – is for a typical neutron reflectometer along the *z* axis normal to the plane of the grating structure. The fractional uncertainty in *Q* is given by

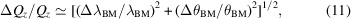

where Δλ_BM_/λ_BM_ = Δ*k*_BM_ / k_BM_, the subscript BM indicating a beam mean or average of the individual λ_M_ or k_M_ mean packet values. The first term on the right-hand side of equation (11) represents the spread in wavelength or wavevector magnitude in the beam (Δλ_BM_/λ_BM_ ≃ 0.01 for a typical reflectometer), and the second term describes the degree of geometrical angular divergence in the beam. As discussed in the preceding sections, it is the latter term that is of interest here. For a given magnitude of *k*_M_, the range of *Q_z_* due to the beam angular divergence, is, in the small-angle approximation, given by

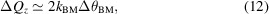

where Δθ_BM_ is the geometrical angular divergence of the monochromated beam defined by a pair of slit apertures of appropriate width and separation distance such that Δθ_BM_ is of the order of a few minutes or seconds of arc (and for glancing angles of incidence θ of the order of a few degrees at most). In practice, the instrumental resolution at the critical edge for total external reflection as well as at the positions of the first few Kiessig fringes (due to the finite thickness of the film bars along the surface normal) is well approximated by equation (11).

As will be demonstrated below, the primary effect of increasing geometrical angular beam divergence is to smear out features of the reflectivity as a function of *Q_z_* (*e.g.* the Kiessig fringes), whereas the observation of either a single or a double critical angle and corresponding plateaus depends upon the transverse coherent extent of an individual neutron wave packet.

**Figure 10:**
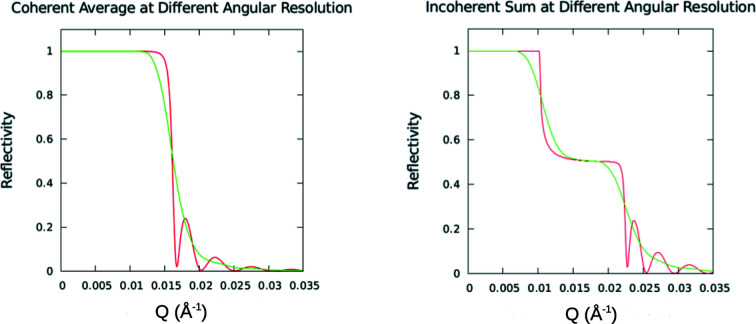
Model calculations of the specular neutron reflectivity as a function of *Q* for different beam angular resolutions in the two limiting cases. In the left-hand plot the transverse dimension of the wavefront is of sufficient extent to completely average over a large enough number of the bars and troughs of the patterned film structure. Conversely, in the right-hand plot the transverse dimension of the wavefront was significantly less than the width of a bar or trough (bar width = trough width). The substrate was taken to be silicon with approximately 950 Å-thick nickel bars deposited on top. Neutrons were taken to be incident from vacuum. Both plots show specular reflectivity curves at two extremes of instrumental angular beam divergence, approximately 3.5 × 10^−5^ and 1.5 × 10^−3^ rad, at a fractional wavelength resolution of 0.01. Despite a difference of a factor of over 40 in the angular divergence of the beam and the consequential rounding of the critical edge and smearing of the film thickness fringes at the broader angular divergences, an unambiguous distinction between the cases for coherent averaging and incoherent sum can be made.

**Figure 11:**
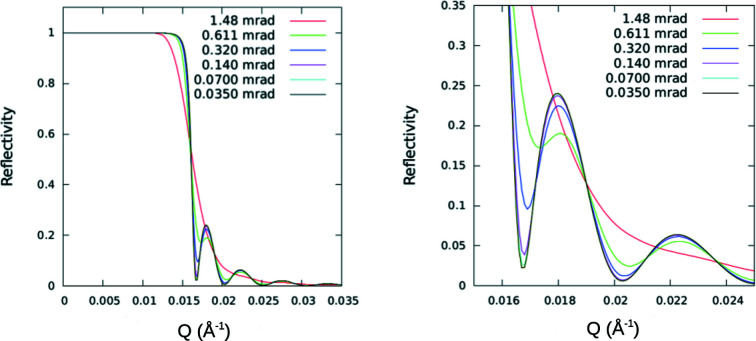
(Left) Model calculation showing a reduction in the Kiessig fringe visibility with broadening beam angular divergence as predicted for the instrumental *Q_z_* resolution perpendicular to the patterned film surface. (Right) Detail of the first two fringes. The geometrical beam angular divergence ranges from 3.5 × 10^−5^ to 1.48 × 10^−3^ rad.

To better illustrate the different roles that the angular resolution of the beam and the finite transverse extent of an individual neutron packet wavefront have on the observed specular reflectivity, model calculations were performed for different instrumental beam resolutions in the two limiting cases: (1) the transverse dimension of the wavefront is of sufficient extent to completely average over a large enough number of the bars and troughs of the structure; and (2) the widths of the bar and trough are each significantly larger than the projected transverse extent of the neutron wavefront (bar and trough widths are equal). The substrate was taken to be silicon with approximately 950 Å-thick nickel bars deposited on top, with neutrons incident from vacuum. Fig. 10 ▸ shows plots for both cases, the coherent average and incoherent sum, at two extremes of instrumental angular beam divergence. These plots clearly demonstrate that the beam angular divergence and associated instrumental resolution along the *z* axis normal to the film surface can be measured separately from the transverse extent of the wavefronts within an individual neutron wave packet. Fig. 11 ▸ shows in more detail the reduction in the Kiessig fringe visibility with broadening beam angular divergence.

It was found in earlier work (Majkrzak *et al.*, 2014 ▸), by measuring the specular reflectivity from a set of repeated-stripe film patterns over a range of different periods, that the transverse dimension of a packet wavefront is of the order of 1 µm. In the present work reported here, more precise measurements of the specular reflectivity were performed on the 10 µm Ni bar width + 10 µm trough width = 20 µm period grating as a function of beam angular divergence (the Ni bars were 950 Å thick). The standard neutron reflectometer configuration was employed, the essential components of which are depicted in Fig. 2 ▸, except that a polycrystalline Be filter was inserted between the HOPG(002) monochromator and neighboring slit (to remove higher-order neutron wavelengths). A guide tube emanating from the liquid hydrogen moderator cold source at the NCNR illuminated the PG. In addition, however, the exit slit prior to the grating sample (which would be located just to the right in Fig. 2 ▸) was positioned in close proximity to the sample to ensure that the beam footprint was fully intercepted by the 75 mm-diameter Si substrate upon which the patterned thin-film structure was deposited, even at the largest beam divergences. A detector was positioned downstream at a scattering angle equal to twice the glancing angle of incidence.

**Figure 12:**
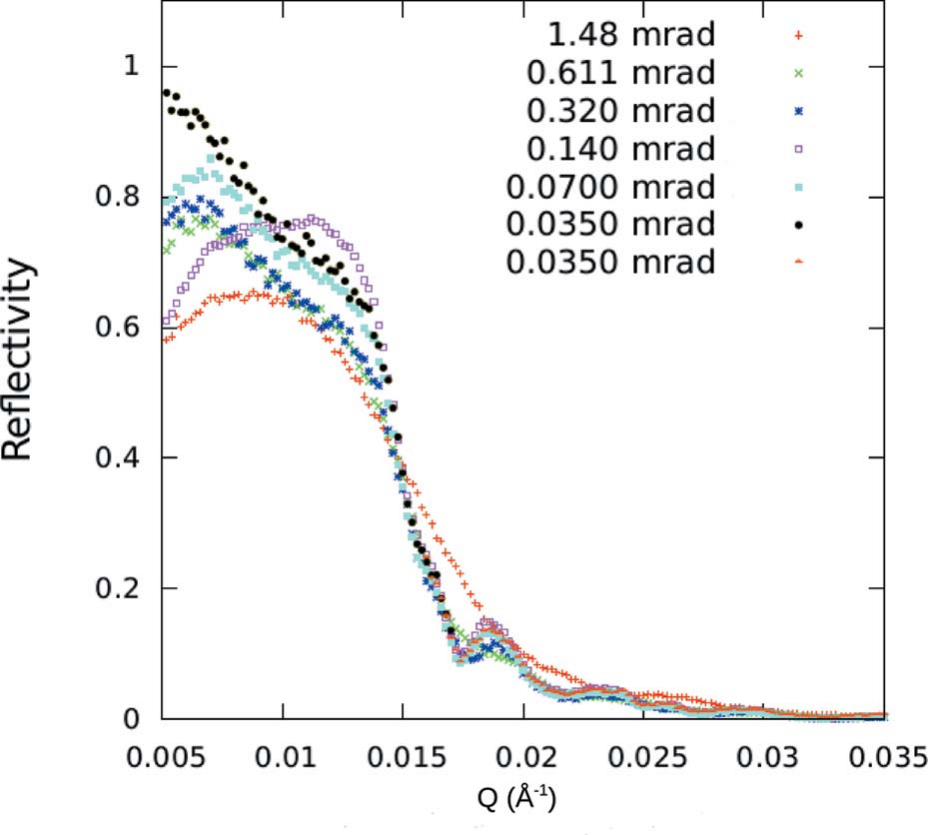
Composite plot of the measured specular reflectivites as a function of beam angular divergence for the 10 + 10 = 20 µm patterned film structure with the mean beam wavevector perpendicular to the grating bars. The values of the angular beam divergence and the corresponding aperture widths are given in Table 4 ▸. Geometrical beam footprint corrections have not been made and the effect of non-specular scattering and other details are discussed in the main text. Also, for clarity, error bars have not been plotted: however, for the data collected at the three coarser values of angular divergence, the uncertainty is approximately three times the size of the symbols, whereas the uncertainty in the data corresponding to the three finer divergences is about six times the symbol size. Although the angular divergence of the incident beam was varied by more than a factor of 40, the individual neutron wave packets in each of the different beams had a transverse extent sufficient to effectively average over a significant number of Ni stripes and intervening troughs, thereby resulting in a single critical value of *Q*_c_ corresponding to the mean value of SLD. Note that, because of the rounding of the critical edge due to beam angular divergence, a single common value of *Q*_c_ is best shown by the nearly identical intersection of all of the separate data sets at approximately a value of half the maximum measured specular reflectivity.

Fig. 12 ▸ is a composite plot of the measured specular reflectivities as a function of beam angular divergence (in all cases Δλ_BM_/λ_BM_ ≃ 0.01) for the 10 + 10 = 20 µm pattern with the mean beam wavevector perpendicular to the grating bars. Geometrical beam footprint corrections have not been made. The slight downturn in reflected intensity below *Q* ≃ 0.0075 Å^−1^ approaching the origin is due to the substrate not intercepting the entire footprint of the beam width (which would require an infinitely long substrate at zero *Q*). Ideally, the specular reflectivity for a homogeneous film layer should plateau at nearly unit reflectivity below the critical angle. However, for the patterned film structure here it dips to about 85% approaching the critical *Q*_c_ because of competing non-specular scattering from the periodic (but inhomogeneous) in-plane patterned film structure. The presence of non-specular scattering was confirmed in scans along the *Q_x_* axis as well as in detector 2θ (or scattering angle) scans at fixed sample or θ angles. This effect is essentially irrelevant to the specular measurements regarding either beam angular resolution or transverse packet dimensions. Moreover, as the angular divergence of the beam increases, the location of the critical angle becomes less well defined. (The values of the angular beam divergence and the corresponding aperture widths are given in Table 4 ▸.)

Although the angular divergence of the incident beam was varied by more than a factor of 40, the individual neutron wave packets in each of those different beams had a transverse extent sufficient to effectively average over a significant number of Ni stripes and intervening troughs, thereby resulting in a single critical value of *Q*_c_ corresponding to the mean value of SLD. On the other hand, it is found that even for the narrowest angular beam divergence the bars and troughs of a stripe pattern with a 20 + 20 = 40 µm period (only twice as long) are not averaged over.

Table 4 ▸ lists typical reflectometer slit widths and beam angular divergences corresponding to the data shown in Fig. 12 ▸, along with the geometrical angular resolution for the incident beam. The geometrical angular widths (FWHM) calculated from the slit widths and their separation distance are typically consistent with measured values to within a few (2–3) per cent accuracy for slit widths of approximately 0.1 mm or greater. Note that if the coarsest instrumental (geometrical) beam resolution listed in Table 4 ▸ had been used in the uncertainty relation [equation (2)] – which would not be appropriate – it would predict the transverse coherent dimension of an incident neutron wavefront Δ*r*_T_ to be 1/2Δ*k*_BMT_ = 0.0269 µm [where Δ*k*_BMT_ represents the distribution of transverse (subscript T) components of mean (subscript M) packet wavevectors in the beam (subscript B), as defined in Table 4]. This value would be far too small to average over the stripes and troughs of the 20 µm-period structure and would be in contradiction to that indicated by the data shown in Fig. 12 ▸. Once again, evidence shows that the packet Δ*r*_T_ is not obtained from the distribution of transverse components of mean packet wavevectors, which defines the geometrical angular divergence of the incident beam {where Δ*k*_BMT_ ≃ *k*_BM_Δθ_BM_ and Δθ_BM_ ≃ arctan[(*W*_1_ + *W*_2_)/(2*L*_12_)]}.

**Figure 13:**
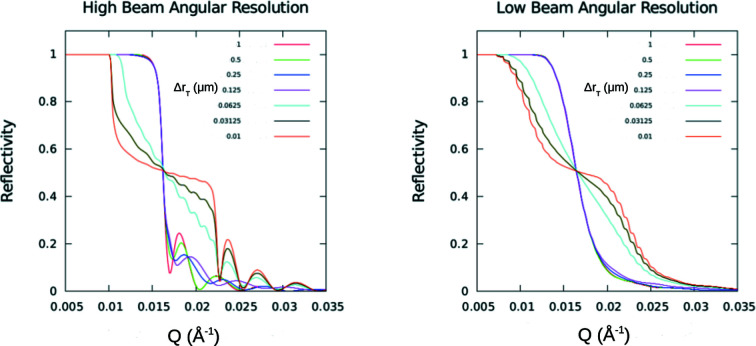
Calculated model specular neutron reflectivity curves about the effective critical angle for a 10 + 10 = 20 µm patterned film repeat (Ni stripes 950 Å thick) with the neutron wavevector perpendicular to the Ni stripes at relatively high (7.0 × 10^−5^ rad, left plot) and low (1.5 × 10^−3^ rad, right plot) instrumental beam angular resolution, for different values of Δ*r*_T_. The glancing angular dependence of the projection given by equation (10), *i.e.* Δ*r*_T_ = *L*sinθ, was explicitly taken into account. Despite the marked difference in instrumental angular beam divergence, the transverse extent of uniform phase of a neutron packet wavefront has a clearly distinguishable effect on the specular reflectivity in the critical angle region in both cases.

Fig. 13 ▸ shows model specular neutron reflectivity curves about the effective critical angle for a striped pattern with neutron wavevector perpendicular to the stripes at two extremes of instrumental beam resolution and where the glancing angular dependence of the projection of Δ*r*_T_, given by equation (10), was explicitly taken into account. To generate the model specular NR curves in this figure, the one-dimensional time-independent Schrödinger equation was solved, employing plane-wave forms in such a way that the effect of the range over which averaging is performed by a wavefront of finite extent was accounted for separately.

The use of striped thin films to determine the transverse extent of wavefronts has also been applied in a related but different manner to study X-ray coherence properties (Lee *et al.*, 2011 ▸; Tolan *et al.*, 1992 ▸, 1994 ▸; Salditt *et al.*, 1994 ▸).

#### 6.2.1. The effect of surface flatness on specular reflection at glancing angles

In preceding sections, measurements of the transverse width of the neutron packet wavefront via diffraction from phase gratings at normal incidence as well as by specular reflection from thin-film striped patterns at glancing angles were described. As might have been noticed, markedly different results between the two methods were obtained. Using phase gratings, in the case of the work reported herein as well as that of others (Treimer *et al.*, 2006 ▸), the uniform transverse extent of the packet wavefront was found to be of the order of tens of micrometres, in contrast to a value of about 1 µm or less in the method involving specular reflection. The cause of this inconsistency was not immediately obvious. After all, the instruments in all cases employed an HOPG(002) pre-monochromator at nearly the same neutron wavelength and with comparable geometrical angular divergences of several seconds of arc, as described earlier. Although the USANS instrument employed by Treimer *et al.* (2006 ▸) also included a multiple-bounce channel-cut Si(111) monochromator and analyzer, the calculations as well as the measurements presented here indicate that the HOPG 002 reflection from a micro-crystal block alone probably suffices to impart to the neutron packet a Δ*r*_T_ of the order of tens of micrometres.

The discrepancy is probably related to the flatness of the supporting Si substrates on which the Ni-stripe thin-film patterns are deposited. In the case of specular reflection from thin-film patterned structures at low wavevector transfer (glancing angles of incidence relative to the reflecting surface), curvature of the underlying support substrate can affect the measurement of the incident neutron packet Δ*r*_T_ and distort that of the specularly reflected one, as shown in previous work (Majkrzak *et al.*, 2014 ▸). On the other hand, for a phase grating etched onto a silicon substrate and oriented perpendicular to the incident beam in the transmission geometry, the geometrical or angular amplification by the sine function is suppressed and the surface flatness requirement to maintain uniform phase across an incident wavefront is thereby relaxed.

As a practical consequence for specular neutron reflectometry studies of layered thin-film structures, the supporting substrate can effectively become a limiting component of the instrumental optics in that its curvature can restrict the surface area over which an incident packet can engage in a coherent specular scattering process. Moreover, the effect of surface curvature in distorting the wavefronts of a reflected packet can have consequences – such as for reflection within neutron guide tubes.

## 7. Measures of coherence

As we have shown, for a single neutron, the region from which coherent elastic scattering can occur is determined by its wave packet. In particular, if the transverse width of an incident wave packet is uniform over the entire extent of a material object, then the wave packet can be coherently scattered as an incident plane wave would be. A beam can be characterized as a collection of individual wave packets of similar size and shape associated with a distribution of packet mean wavevector directions that define a geometrical angular divergence. This angular divergence represents a component of the instrumental beam resolution which limits the ability to resolve the intrinsic structure of a diffraction or specular reflection pattern.

Both the collective distribution of packet mean wavevectors in the beam and the set of basis wavevectors within each individual packet limit what can be observed about the intrinsic diffraction pattern for a given object. However, it is the coherent superposition of basis states which compose an individual packet wavefunction alone that determines the transverse extent over which a particular wavefront is of sufficiently uniform phase to interact simultaneously with scattering material in a coherent manner (thereby creating a superposition of reflection amplitudes).

In the following sub-section we consider a well known theory of partial coherence developed for light optics – but applicable to neutrons as well – with the aim of making clearer the sometimes confusing issue regarding the differences between plane-wave beams and beams of wave packets.

(Because of the relatively large number of different quantities that are involved in this section and the associated Appendix *B* [App appb], a glossary of variables and their definitions is given at the end of Appendix *B* [Sec secb3].)

### 7.1. Theory of partial coherence

A theory of partial coherence was developed originally for ordinary light optics by Mandel & Wolf (1965 ▸, 1995 ▸) and others and has since been applied to both X-rays (Sinha *et al.*, 1998 ▸, 2014 ▸; Kaganer *et al.*, 2001 ▸) and neutrons (Gähler *et al.*, 1998 ▸; Felber *et al.*, 1998 ▸; de Haan *et al.*, 2008 ▸, 2010 ▸). It is applicable to plane wave as well as paraxial type wavefunction forms. One of the core tenets of this partial coherence theory is to adopt a two space–time point correlation function as a measure of coherence. Although such a measure of coherence can be a useful tool for certain aspects of the analysis of diffraction data, by itself, it can be incomplete or ambiguous in regard to the measure of the spatial extent of a packet wavefront over which the phase can be considered to be of sufficient uniformity. This can be illustrated by a classic textbook example of a two-dimensional incoherent line source of plane waves (Born & Wolf, 1975 ▸; Hecht, 1998 ▸) compared with that for wave packets – as will now be shown.

**Figure 14:**
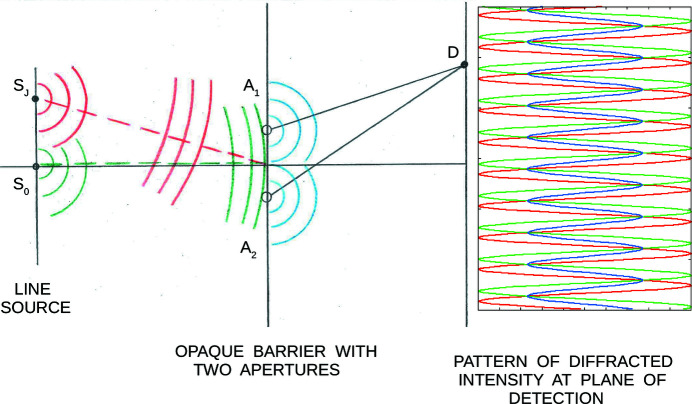
Young’s experiment for creating an interference pattern in which two apertures are illuminated with quasi-monochromatic radiation from a temporally and spatially extended incoherent source (the waves from the two source points shown are emitted independently of one another with a random phase difference). In this two-dimensional illustration, the radiation is emitted as circular waves from each point on a line. The distances between source and apertures and between apertures and detection line are great enough that the Fraunhofer or far-field limit is a valid approximation for describing the scattering. At the location of the apertures A_1_ and A_2_, the circular wavefronts are nearly planar. Any single-aperture diffraction that might occur and modulate the two-slit interference pattern plotted on the far right-hand side of the figure is neglected since it is not relevant to the arguments made concerning the role of the mutual coherence function and fringe visibility discussed in the text (this is equivalent to both single-aperture widths approaching zero). The red and green patterns of intensity plotted on the right correspond to two point sources, one at the origin S_0_ and the other off the horizontal axis of symmetry at S_1_, respectively. The blue curve results upon adding these two single-source-point intensity distributions together. Because of the translational offset between the two single-point patterns, a reduced ‘fringe’ visibility or diminished instrumental resolution results.

According to this theory of partial coherence, one way of defining coherence is to quantify it through an association with the cross correlation function

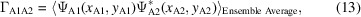

referred to as the mutual coherence function (MCF). The MCF is constructed as follows. First, the wavefunction Ψ_A1_(*x*_A1_, *y*_A1_) for a single wave emanating from a given point on an extended source of incoherent points of emission – as shown in Fig. 14 ▸ – is evaluated at some distant point A_1_. The product of this wavefunction and its complex conjugate evaluated at a different point A_2_ is then integrated over all possible realizations of paths from the extended source through points A_1_ and A_2_, thereby forming an ensemble average for all the radiation emitted by the source. The integrand is a measure of the phase difference between the two points A_1_ and A_2_ for the wave from each of the possible source points. The mutual coherence function incorporates the statistical nature of the scattering process wherein the diffraction pattern emerges after a sufficient number of scattering (diffraction) events are recorded through the averaging over all possible trajectories a neutron can take from a source point to the diffracting object. The normalized form of the mutual coherence function γ_A1A2_, named the complex degree of coherence, is given by

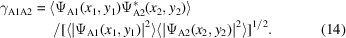

The modulus of γ_A1A2_ can be shown (*e.g.* Born & Wolf, 1975 ▸) to be identical to the fringe visibility *V* that would be observed in an interference pattern (equivalent to Young’s two-slit diffraction experiment) as given by

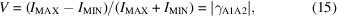

where (0 ≤ *V* ≤ 1). Fig. 14 ▸ illustrates pictorially what the complex degree of coherence or fringe visibility measures. A more comprehensive discussion of the derivation of the mutual coherence function and complex degree of coherence is given in Appendix *B* [App appb] along with a glossary of the definitions of the relevant quantities involved.

In brief, the principal information contained in γ_A1A2_ is the degree to which the features of an intrinsic diffraction pattern can be resolved when the diffracting object is illuminated by an extended source of independent, incoherent emitters – given that the wave emanating from each source point is by itself perfectly coherent and for the case of plane waves of infinite transverse extent. No explicit information about the transverse dimensions of the wavefronts is contained in the complex degree of coherence. The diffraction pattern which would have been observed for two infinitesimally narrow slits positioned at points A_1_ and A_2_ results from adding together the diffracted intensities contributed by each one of the separate and independent source points that are incoherently related to one another. Nonetheless, as reproduced in Appendix *B*, a ‘coherence length’ has been conventionally defined in terms of the fringe visibility represented by the function γ_A1A2_ as it approaches zero value.

**Figure 15:**
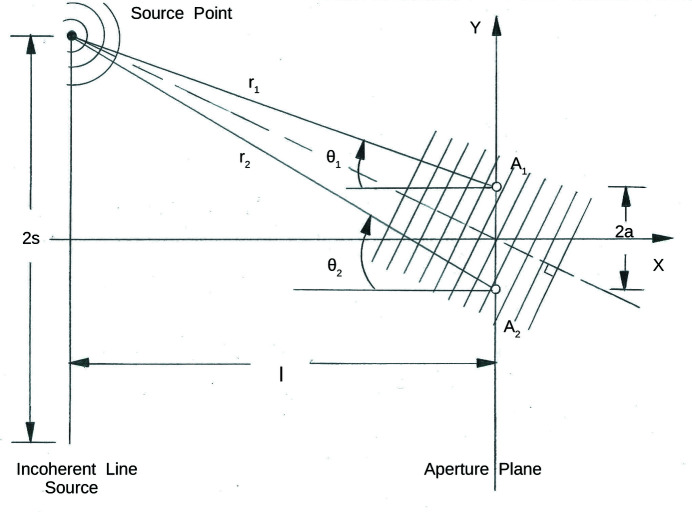
The relevant part of Fig. 14 ▸ is redrawn in more detail here for reference to the discussion in the text regarding the calculation of the mutual coherence function and complex degree of coherence.

For the specific example just given above, in which the incoherent line source is of uniform emittance [see Appendix *B* [App appb] and the derivation therein leading to equation (31) or (16) below],

*[plane waves, uniform source]*




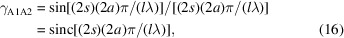

where 2*s* is the width of the line source, 2*a* is the distance between points A_1_ and A_2_, *l* is the distance between the source line and points A_1_ and A_2_ (see again Appendix *B* [App appb] and Fig. 15 therein), and λ is the wavelength of the radiation. Setting the argument of the sinc function to π at which the first zero occurs (and beyond which the features of the interference pattern are significantly and progressively further diminished) defines a limit on the maximum width the source can be before resolution of diffraction pattern features is no longer possible. This condition gives the relation (once again, consult Appendix *B* [App appb] for details)

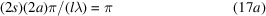

or

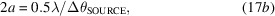

where Δθ_SOURCE_ ≃ *s*/*l*. Since *k* = 2π/λ, the quantity *k*Δθ_SOURCE_ is a measure of the width of the distribution describing an uncertainty in wavevector components transverse or normal to the mean direction of propagation. For a given wavelength and aperture spacing and a fixed distance between source and apertures, the fringe visibility decreases with increasing source size – or, equivalently, with an increasing range of the angular distribution of trajectories of wavevectors directed from the source points towards the apertures. That is, the greater the angular divergence of the incident beam, the poorer the resolution of the features of the interference pattern. To resolve a spatial dimension of the order of 2*a* (the separation of the two points A_1_ and A_2_), the instrumental beam resolution must be of the order of *k*Δθ_SOURCE_. The distance 2*a* is conventionally taken to be a measure of coherence. But it is actually proportional to the angular divergence of the beam and does not directly provide any explicit information about the width of the wavefront. Rather, it is implied by the assumption of a plane-wave form that every wavefront emitted from any single point on the extended source has a phase uniform to within one wavelength at the location of the two points a distance 2*a* apart on the diffracting sample object.

As a second example, suppose that the evaluation of γ_A1A2_ performed above is repeated for a similar incoherent source which is identical in all respects except that, instead of a line source of uniform emittance, the intensity distribution of the point sources is Gaussian, centered on the origin of the *y* axis. In this case it is straightforward to show that (see Appendix *B* [App appb] for details)

*[plane waves, Gaussian source]*


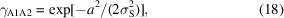

where σ_S_ is the standard deviation of the Gaussian source intensity distribution representing the width of the source line along the *y* axis, analogous to the half-width *s* in the previous example above. As shown in Appendix *B* [App appb], σ_S_ = 1/σ_*k*_, where σ_*k*_ is the corresponding standard deviation of a Gaussian distribution of transverse components of wavevectors and is directly related to the beam angular divergence Δθ_SOURCE_ and wavevector **k**. In this case, the relationship between 2*a* and the source width can be obtained, for instance, by setting γ_A1A2_ = 0.5 (at the HWHM) so that

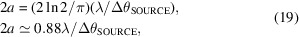

which is comparable to the previous result given by equation (17).

As one more (particularly revealing) example, suppose that the truncated wavefunction representing the elongated wave packet described by equation (7) is chosen for the calculation of the complex degree of coherence γ_A1A2_ instead of a plane wave. Again, assume the Gaussian distribution of emitter strengths along the extended source line as in the example immediately above. Now, however, each isotropic point emitter which produced a nearly plane wave at A_1_ and A_2_ is replaced by a micro-crystal block that – through the wavelength- and angle-selective Bragg diffraction process – produces a truncated plane wavefront [similar to that described by equation (7)] directed through those two points. Following exactly the same procedure as for the previous two examples, it is found (refer, once again, to Appendix *B* [Aff b] for calculation details) that

*[wave packet, Gaussian source]*


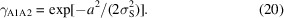

The term exp[−*y* ^2^/(2σ^2^_*y*WP⊥_)] of equation (7), representing the coherent superposition of wave amplitudes for the basis states of the individual neutron packet wavefunction, cancels out of the expression for the complex degree of coherence. What remains in equation (20) is an expression describing the incoherent distribution of mean wavevectors in the beam, which corresponds to the instrumental angular divergence. This expression is equivalent to that obtained for the plane wavefunction leading to the result given by equation (18). This result is consistent with what the complex degree of coherence and fringe visibility represent, as discussed earlier. No information about wavefront width, explicit or otherwise, is contained in γ_A1A2_.

On the other hand, the mutual coherence function Γ_A1A2_ – which is essentially the un-normalized complex degree of coherence [see equations (13) and (14)] – does contain parameters characterizing the width of the packet wavefront. As derived in Appendix *B* [App appb], Γ_A1A2_ for the wave packet example is given by

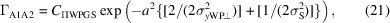

where the standard deviations σ_*y*WP⊥_ and σ_S_ correspond to an individual wave packet width and the source width, respectively. [The factor C_ΠWPGS_ contains other normalization constants for the Gaussian wave packet function (‘WP’) and Gaussian source (‘GS’).] Note that, although the mutual coherence function includes the widths of both packet and source distributions, the two are combined in the width of one composite Gaussian distribution. Any given value of the width of that composite distribution (as characterized in the argument of the exponential) can be composed of different combinations of the separate packet and source widths σ_*y*WP⊥_ and σ_S_ and, therefore, cannot represent a single unique measure of coherence ‘length’.

In summary, the complex degree of coherence primarily describes the degree to which the incoherent addition of intensity contributions from different source points diminishes the ability to resolve features in the intrinsic diffraction pattern for a given object (which would have been completely resolved if illuminated by a single ideal point source). It quantifies the effect of beam angular divergence on the instrumental resolution. The complex degree of coherence is not a direct measure of the transverse uniformity of a wavefront in a packet associated with any one individual quantum particle in a beam originating from any particular source point.

A full analysis of instrumental effects in specular reflection consequently requires knowledge of two separate measures, one corresponding to the coherent transverse extent of individual packet wavefronts and the other associated with the geometrical angular divergence of the mean wavevectors of the incoherent collection of packets composing the beam. But as has already been described in Section 6 above, there are means to obtain information about the effective transverse wavefront dimensions nearly independently of the geometrical beam angular divergence.

## 8. Discussion

In the model employed in the present work, each neutron in a beam is represented by an independent wave packet function. The principles of standard quantum mechanics theory constrain what form a wavefunction can take to describe a pure state associated with a single Fermi quantum particle. Berk (2018 ▸) shows that a fermion wave packet appropriate for the description of scattering problems (and which may be constructed, for instance, of a linear superposition of pure plane-wave states) represents a pure quantum state of a single particle. On the other hand, mixed states can be constructed to describe beams of independent particles created by appropriate incoherent sources (Berk, 2018 ▸). The corresponding differences between pure and mixed states in their respective formal representations as statistical operators or ‘density matrices’ is also discussed by Berk (2018 ▸). The interpretation of the representation of beams in terms of pure and mixed states, as considered, for example by Ballentine (1988 ▸), has been further critically examined more recently by Berk (2018 ▸).

## 9. Conclusion

The full analysis of specular neutron reflectivity measurements from surfaces or interfaces with inhomogeneous in-plane density distributions requires knowledge of the transverse extent of neutron packet wavefronts over which the phase is of sufficient uniformity. A plane-wave representation of the neutron is not always adequate, and wave forms with limited transverse extent that more accurately describe how the neutron wavefunction is prepared in the instrument need to be adopted.

There are two distinct distributions of wavevectors associated with a beam of freely propagating neutrons as prepared in a typical scattering instrument. One consists of the wavevectors of the components of a coherent superposition of basis states that constitute each individual neutron’s corresponding wave packet function – *i.e.* the distribution of wavevectors associated with a single neutron that define its transverse coherent extent. The other is made up of an incoherent collection of mean packet wavevectors associated with the entire ensemble of individual neutrons composing a beam – *i.e.* the distribution of the individual neutron packet mean wavevectors associated with a geometrically defined beam angular divergence.

Both the distribution of the mean wavevectors of all of the neutron packets in the beam and the wavevector components of the superposition of basis functions within an individual packet limit what can be observed about the intrinsic diffraction pattern for a given object. However, it is the transverse spatial extent of packet wavefronts – over which the phase is sufficiently uniform – alone that determines the area over which a coherent scattering process with matter can occur. This picture is consistent in principle with the formal tenets of the standard quantum theory for describing scattering.

Although the exact shape or form of the neutron packet may not be known, approximate models which limit the transverse extent of the packet, for example, a packet with truncated nearly planar wavefronts, or one as described by equation (7), may provide for a sufficiently accurate analysis of specular reflectivity in practical cases. Other wavefunctions can of course be adopted depending on how a neutron is prepared on a given instrument.

Because the same monochromating and collimating devices, such as crystals and slits, may shape each individual neutron wave packet as well as defining the distribution of mean packet wavevectors of the neutrons composing a beam, it might not appear possible to avoid conflating the two effects. However, it is demonstrated that the two distinct distributions – one consisting of the transverse components of the collection of packet mean wavevectors in the beam and the other made up of the transverse components of basis wavevectors intrinsic to each individual wave packet – can be distinguished from one another experimentally in practice.

In the transmission phase-grating measurements reported on here, the effective transverse coherent extent of a packet wavefront was determined to be approximately 24 µm. However, this result pertains to a particular instrumental configuration, *i.e.* the wavefront characteristics depend on the specific way a neutron packet is formed within a given instrument. It would be expected, for example, that the result reported here would differ from that which would be obtained for a time-of-flight instrument not employing a crystal monochromator.

Moreover, it is also shown that the shape of a scattering object under study, for example, a thin-film sample supported by a substrate that deviates from perfect flatness, can also affect the transverse uniformity of a neutron packet wavefront, thereby, in effect, causing the scattering object to act as part of the instrument optics.

## Figures and Tables

**Table 1:** Results of a Huygens–Fresnel (HF) construction for a variety of pertinent aperture widths and distances to a plane of observation for diffraction of an incident plane wave of 5 Å wavelength by a two-dimensional slit aperture The real part of the reflection amplitude as well as the intensity distribution at a plane of observation a distance *S* from the aperture are plotted in Fig. 3 ▸ for two of the cases listed in the table.

*W* (Å)	*S* (Å)	Γ_FWHM_ (Å) (intensity distribution)		Δ*r*_T_ (Å)	Δθ (rad)
Slit width	Slit-to-detector distance	Via HF construction	Fraunhofer limit (analytic)†	HF amplitude	≃ Γ/*S*
10^4^ (1 µm)	0.5 × 10^10^	2.48 × 10^6^	2.50 × 10^6^	2.19 × 10^5^ (21.9 µm)	5.0 × 10^−4^
10^5^ (10 µm)	0.5 × 10^10^	2.53 × 10^5^	2.50 × 10^5^	2.18 × 10^5^ (21.8 µm)	5.0 × 10^−5^
10^6^ (100 µm)	0.5 × 10^10^	8.92 × 10^5^	–	9.26 × 10^5^ (92.6 µm)	1.8 × 10^−4^
10^5^ (10 µm)	2.5 × 10^10^	1.25 × 10^6^	1.25 × 10^6^	4.63 × 10^5^ (46.3 µm)	5.0 × 10^−5^
10^6^ (100 µm)	2.5 × 10^10^	7.74 × 10^5^	–	7.41 × 10^5^ (74.1 µm)	3.1 × 10^−5^

† For the Fraunhofer limit, analytic *y*_MIN_ ≃ *S*λ/*W*, which is approximately Γ_FWHM_.

**Table 2:** Comparison of the differences between the magnitudes of the widths of the transverse wavevector component distributions associated with an individual wave packet and that of the packet mean wavevectors contained in the beam *N* is the number of periods that a wavefront of width Δ*r*_T WP_ would cover for a grating having a 2.4 µm repeat distance. The last column on the right gives the ratios of the FWHM of the packet mean wavevector distribution Δ*k*_MT(BEAM)_ divided by an individual packet’s component basis wavevector distribution Δ*k*_T WP_. (The nominal neutron wavelength is 5 Å.)

Incident beam angular divergence Δα				Δ*k*_MT(BEAM)_	*N*	Δ*r*_T WP_	Δ*k*_T WP_	Δ*k*_MT(BEAM)_/Δ*k*_T WP_
(rad)	(°)	(′)	(′′)	(Å^−1^)		(µm)	(Å^−1^)	
1.75 × 10^−5^	0.001	0.06	3.6	2.19 × 10^−5^	2	4.8	1.04 × 10^−5^	–
3.50 × 10^−5^	0.002	0.12	7.2	4.38 × 10^−5^	2	4.8	1.04 × 10^−5^	4.21

1.75 × 10^−5^	0.001	0.06	3.6	2.19 × 10^−5^	4	9.6	5.20 × 10^−6^	–
3.50 × 10^−5^	0.002	0.12	7.2	4.38 × 10^−5^	4	9.6	5.20 × 10^−6^	8.42

1.75 × 10^−5^	0.001	0.06	3.6	2.19 × 10^−5^	30	72.0	6.90 × 10^−7^	–
3.50 × 10^−5^	0.002	0.12	7.2	4.38 × 10^−5^	30	72.0	6.90 × 10^−7^	63.5

Δ*r*_T WP_Δ*k*_T WP_ = 1/2 or Δk_T WP_ = 1/(2Δr_T WP_), Δ*k*_MT(BEAM)_ = *k*_M_sin(Δα), and Δα is the FWHM of the angular divergence distribution of the incident beam.

**Table 3:** Values of the critical wavevector *Q*_c_ for relevant materials – Q_c_^2^ = 16πρ

Material	*Q*_c_ (Å^−1^)
Ni (unmagnetized)	0.0217
Si	0.0102
50% Ni + 50% Si (by volume)	0.0170
50% Ni + 50% vacuum (by volume)	0.0154

**Table 4:** Typical reflectometer slit widths and geometrical angular divergences for the incident beam corresponding to the data shown in Fig. 12 ▸ The angular widths (FWHM) calculated from the slit widths and their separation distance are typically measured to be consistent to within a few (2–3) per cent accuracy.

*W*_1_ (mm)	*W*_2_ (mm)	*L*_12_	Δθ_BM_ (rad)	2Δ*k*_BMT_ ≃ 2*k*_BM_Δθ_BM_ (Å^−1^)
0.05	0.05	1429	3.50 × 10^−5^	8.80 × 10^−5^
0.10	0.10	1429	7.00 × 10^−5^	1.76 × 10^−4^
0.20	0.20	1429	1.40 × 10^−4^	3.52 × 10^−4^
1.00	0.10	1719	3.20 × 10^−4^	8.04 × 10^−4^
2.00	0.10	1719	6.11 × 10^−4^	1.54 × 10^−3^
5.00	0.10	1719	1.48 × 10^−3^	3.72 × 10^−3^

## Appendix A Instrumental specifications

At the NIST Center for Neutron Research, the reflectometer MAGIK (formerly named AND/R prior to relocation; Dura *et al.*, 2006 ▸) was specifically configured for the measurements reported herein as follows. A composite HOPG monochromator was situated in a gap on the NG-D neutron guide tube and was aligned to Bragg reflect neutrons with wavelengths of approximately 5.00 ± 0.01 Å. The distance between the monochromator and the first slit aperture downstream was approximately 908 mm (in between which was positioned a polycrystalline block of Be cooled to liquid nitro­gen temperature to remove higher-order wavelength neutrons). The distance between the first and the second slit was *L* = 863.6 mm. Both slits had a nominal width *W* = 0.025 mm. Each slit aperture extended along the vertical direction – out of the plane of Fig. 2 ▸ – by approximately 25 mm and was defined by a pair of parallel absorbing Cd masks, 1 mm thick, with accurately machined edges. An identical ‘detector’ slit was situated in front of a ^3^He detector tube a distance of 1651 mm away from the second slit. The detector slit could be scanned along a direction perpendicular to an axis defined along the center of the two collimating slits upstream. The geometrical divergence limits ±ɛ of the pair of collimating slits are given by tanɛ = *W*/*L*. For the numerical values given above, ɛ = ±2.895 × 10^−5^ rad (or 1.659 × 10^−3^° = 9.952 × 10^−2^′ = 5.97′′). Projecting back through the pair of slits toward the HOPG monochromator upstream, the width viewed at the monochromator position was approximately 7.76 × 10^−2^ mm (although a width of about 25 mm across the surface of the monochromator illuminated the entrance to the first slit with a relatively uniform – spatially and in angle – flux of quasi-monochromatic neutrons).

## Appendix B The mutual coherence function and complex degree of coherence

(Note that, because of the relatively large number of different quantities that are involved in this appendix and in Section 7 of the paper to which it refers, a glossary of variables and their definitions is given at the end.)

The mutual coherence function is a measure of the degree to which the spatial extent of an incoherent radiation source diminishes the ability to resolve certain features of a diffracted intensity pattern. This composite pattern formed from an extended source is the result of a summation of distinct intensity contributions from each point source component on the extended source. Each of these intensity contributions is created in the first place by a coherent process involving the superposition of component amplitudes originating from a given object illuminated by a single point source (at a sufficient distance that the wavefronts have become effectively planar). Subtle distinctions between quantities representing a superposition of amplitudes in contrast to those which are a summation of intensities are important.

The basic meaning of the mutual coherence function and related quantities is typically illustrated through the simple example of how the interference pattern produced by a pair of apertures – illuminated by a perfectly monochromatic and spatially coherent point source of light (Young’s experiment) – differs from that which arises if the waves are quasi-monochromatic and emanate from an extended incoherent source as depicted in Fig. 14 ▸ (the slit widths are taken to be zero so that no modulating envelope due to diffraction by each of the individual slits appears in the intensity plot of the figure). This classic experiment is described in numerous texts on optics, but we follow more closely the descriptions given by Born & Wolf (1975 ▸), Hecht (1998 ▸) and Mandel & Wolf (1995 ▸).

To begin, several simplifying assumptions and approximations are made that are valid for the specific case of interest at hand, namely, the elastic scattering of neutrons that originated in a temporally and spatially extended incoherent source such as a liquid hydrogen moderator at a continuous flux reactor. We will also ignore neutron polarization and consider only scalar wavefunctions as opposed to spinors (analogous to neglecting the polarization of electromagnetic radiation). The usable source intensity can be considered, for the sake of argument, sufficiently weak that only one neutron at a time interacts with the diffracting object and the measuring instrument (diffractometer or reflectometer). In typical circumstances, a single neutron may pass from source to diffracting object to detector before another neutron arrives as discussed in the main text. This means that any interference phenomena resulting from the interaction of a single neutron and a diffracting object arises strictly in terms of the component basis states of which the wave packet representing that particular neutron is composed. It is also a well established quantum phenomenon that the observation of the diffraction pattern is stochastic in nature, requiring a sufficient number of apparently random detections of individual neutrons across a range of angular or spatial positions to reveal the underlying form of the pattern.

Further, it is assumed that the neutrons are quasi-monochromatic. This means that each corresponding wave packet possesses a temporal coherence length (which is often referred to as a longitudinal spatial coherence length along the mean direction of propagation) that is significantly larger than its transverse coherence length (perpendicular to the mean direction of propagation) – similar in form to that which we adopted in preceding sections. Here we are primarily concerned with the transverse coherence length or, more accurately, the transverse coherent extent of the wavefronts within the neutron wave packet over which the phase is constant and can thereby give rise to coherent scattering from an effective average material density. We also assume stationarity and ergodicity, *i.e.* that any time average over a collection of similar neutron quantum particles successively interacting with the scattering object is time independent and that the time average is essentially equivalent to the ensemble average, respectively.

With the simplifications and assumptions so stated, consider again the diffraction arrangement schematically represented (in two dimensions) in Fig. 14 ▸. Let the distances between source and opaque barrier (including apertures) and detector plane be sufficiently large and the source width small enough that the far-field or Fraunhofer limit is a valid approximation for describing the diffraction. We first consider the case where circular wavefronts emanate from the source at a point S_0_ along the axis of symmetry, equidistant from each aperture, and travel towards the opaque barrier, becoming nearly planar along the way.

At the barrier, the two apertures act as coherent secondary sources which radiate circular waves that subsequently interfere at the detector plane to produce a well known interference pattern of detected intensity – once an adequate number of single neutrons are emitted and diffract in similar fashion. (As indicated previously, because the slit widths are taken to effectively approach zero, the modulating envelope due to diffraction by each of the individual slits does not appear in the intensity plot of the figure). If all the neutrons were emitted from the same central source point, the diffraction pattern would be perfectly resolved and the ‘fringe’ visibility *V* defined in terms of the normalized contrast (difference) between intensity maximum *I*_MAX_ and minimum *I*_MIN_ (*i.e.* the minimum immediately adjacent to the maximum) would be greatest and equal to unity:

Note that the central maximum of the pattern coincides with the position of the horizontal symmetry axis.

Consider next a wave train emanating from another source point S, one that is off the symmetry axis and not equidistant from either aperture. Once again, the two apertures act as secondary sources of circular waves that produce a similar diffraction pattern – but one which is now, on the whole, translationally shifted along the vertical detector axis relative to that associated with the central source point, as shown in Fig. 14 ▸. The sum of the two intensity contributions is also shown in Fig. 14 ▸ and clearly indicates a loss of fringe visibility or resolution as defined by equation (22). In effect, extending the source obscures the interference pattern to some degree relative to what would have been observed with a single point source. This does not imply, however, that a wavefront of the radiation emanating from any one point source has a diminished transverse extent over which the phase is uniform.

The resultant neutron wavefunction Ψ_D_ (here the neutron wavefunction replaces the scalar electric field in an analogous description for visible light) at a point D on the detector plane is the sum of the two waves that emanated from each of the two secondary source points or apertures A_1_ and A_2_ at that point D and is given by

where *t* is a point in time, *v* is the modulus of the phase velocity of the wave, and the (time-independent) coefficients *C*_A1_ and *C*_A2_ account for changes in the wavefunctions that depend upon which aperture the respective component emanated from. In other words, equation (23) says what the wavefunction or field amplitude is at any given position and time on the detector in terms of what the wavefunctions at apertures A_1_ and A_2_ were at earlier times *t*_1_ = *r*_1_/*v* and *t*_2_ = *r*_2_/*v*, respectively.

Ultimately, what is observed on the detector is an intensity pattern acquired over a finite time period τ which is taken to be long in comparison to the coherence time (which is simply related to the longitudinal or temporal coherence length that was discussed earlier). The net intensity ^Σ^ *I*_D_ is obtained by averaging over the finite time interval T and accounts for neutrons emanating from every possible point on the extended source. This average is denoted by

Substituting the explicit expression for Ψ_D_ given in equation (23) into equation (24), expanding, changing variables to τ = *t*_2_ − *t*_1_ (imposing the condition of stationarity), and identifying quantities *I*_D1_ and *I*_D2_ as corresponding to the intensities which would be obtained at point D if only either aperture A_1_ or A_2_ alone had been open, respectively, we obtain [see, for example, Section 12.3 of Hecht (1998 ▸)]

where Γ_A1A2_(τ) = 〈Ψ_A1_(*t* + τ)Ψ_A2_^*^(*t*)〉_T_, Γ_A1A1_(0) = 〈|Ψ_A1_(0)|^2^〉_T_ and Γ_A2A2_(0) = 〈|Ψ_A2_(0)|^2^〉_T_.

The quantity Γ_A1A2_(τ) in the interference term which arises upon superposition of the waves is a two space–time point cross-correlation function that is commonly referred to as the mutual coherence function (Mandel & Wolf, 1995 ▸). The argument of the Re part of the function in the last term on the right-hand side of equation (25), γ_A1A2_(τ), is called the normalized mutual coherence function or the complex degree of coherence. Its modulus can be shown (Mandel & Wolf, 1995 ▸) to be identical to the fringe visibility given by equation (22) [see also Hecht (1998 ▸), Section 12.3], which varies from zero (for complete loss of contrast) to unity (for the optimum contrast possible as would be obtained with a perfectly spatially coherent monochromatic single-point source). For values in between zero and one, the diffraction pattern manifests some degree of partial fringe visibility.

Up to this point in the discussion, no explicit form for the wavefunction Ψ(*r*, *t*) in the formula for the mutual coherence function and complex degree of coherence has been assumed. Although localized wave packet functions are ultimately of interest, it happens that a circular (in two dimensions) or spherical (in three dimensions) wave, which at a sufficient distance from its point source is effectively planar, suffices for describing the fundamental meaning of the complex degree of coherence. (However, in three dimensions it is necessary to consider the normalization involved in calculating absolute intensities properly.) We can take Ψ(*r*, *t*) to play either the role of the electric field intensity for unpolarized electromagnetic radiation or the wavefunction of an unpolarized neutron. As it turns out, to relate the fringe visibility or instrumental resolution to the relevant characteristics of the radiation source, it is necessary only to evaluate the informational content of the normalized mutual coherence function or complex degree of coherence γ_A1A2_(τ). The mathematical details are as follows.

The relevant part of Fig. 14 ▸ is redrawn in more detail in Fig. 15 ▸. Rather than computing the time average associated with the mutual coherence function, we will invoke ergodicity and perform the equivalent alternative ensemble average over all possible realizations of the neutron waves emanating from the extended source.

Let the wavefield emanating from each point on the extended source be circular and far enough from the apertures that the curvature of every wavefront at the location of the apertures can be approximated by a straight line (plane-wave-like in the Fraunhofer limit). The wavefunction in this (two-dimensional case) is given by

where *k_x_* and *k_y_* are the Cartesian components of the neutron wavevector. The quantity that we need to calculate explicitly is

where the ensemble average 〈〉 is carried out over the entire line source from *y* = −*s* to *y* = +*s*. It is assumed that the source emits a uniform distribution of waves at all positions along the line (parallel to the *y* axis). The neutron wavevector direction is specified by the angles θ_1_ and θ_2_ for positions A_1_ and A_2_ on the *y* axis at +*a* and −*a* – where the wavefunctions are evaluated – as indicated in Fig. 15 ▸. The parameters *r*_1_ and *r*_2_ specify the corresponding distances between a particular source point on the line and the points A_1_ and A_2_. The distance between source line and aperture line along the horizontal *x* axis is denoted by *l*. An integration along the source line can be straightforwardly parameterized by expressing the *x* and *y* components of the wavevector, *k_x_* and *k_y_*, in terms of θ_1_, θ_2_, *l* and *a*. Note that the plane wavefronts are approximated to be perpendicular to the line between the origin and the source point – which should be valid for the relatively small angles typically encountered. The essential requirement is to properly account for the phase difference between the two points A_1_ and A_2_. We can write

where we have made the substitutions *k*_*y*1_ = *k*sinθ_1_, *k*_*y*2_ = *k*sinθ_2_, tanθ_1_ = [(*Y* − *a*)/*l*] ≃ sinθ_1_ ≃ θ_1_ and tanθ_2_ = [(*Y* + *a*)/*l*] ≃ sinθ_2_ ≃ θ_2_, and where *Y* has been defined as the position along the vertical line source axis. Then

where *k* = 2π/λ has been substituted, in which λ is the neutron wavelength. Performing similar integrations we obtain

so that

This result is equation (16) in the main text. γ_A1A2_ can be explicitly related to the fringe visibility *V* defined in equation (22) (for the case of the two-slit interference pattern) [see, for example, Hecht (1998 ▸), ch. 12]:

where 0 < |γ_A1A2_| < 1 (a well known result in light optics). Thus, the visibility of a diffraction pattern can be predicted in terms of what would be observed for scattered radiation emanating from a hypothetical pair of points on an object a distance 2*a* apart when illuminated by incident radiation from all of the points on a primary spatially extended incoherent source. For any specified degree of visibility *V* between zero (completely unresolved) and one (maximum resolution), the complex degree of coherence predicts the lateral dimension 2*s* of the primary incoherent source.

The complex degree of coherence or fringe visibility implies that resolution of the features in the interference pattern depends upon the source size (2*s*), the aperture spacing (2*a*), the distance between source line and aperture line (*l*), and the wavelength (λ). For a given wavelength and aperture spacing and a fixed distance between source and apertures, the fringe visibility decreases with increasing source size – or, equivalently, with an increasing range of the angular distribution of trajectories of wavevectors directed from the source points towards the apertures. That is, the greater the angular divergence of the incident beam, the poorer the resolution of the features of the interference pattern.

However, this relationship between fringe visibility or the complex degree of coherence and geometrical angular beam resolution does not imply that the nearly planar wave train emanating from any one source point by itself creates a less-resolved diffracted intensity pattern – it is simply hidden among the similar but shifted patterns contributed by all the other source points which are emitting at the same time. It is this summation of the intensity contributions from a collection of such source points on the extended source line that causes the resolution of the net resulting pattern to be effectively diminished. In this sense, the complex degree of ‘coherence’ – as shown to be associated with the fringe visibility – can be thought of more as a measure of the degree to which increasing the spatial extent of the source effectively obscures the underlying pattern that would have been better resolved by restricting the source size to one point. Once again, this calculation of the mutual coherence function and complex degree of coherence was performed, as typically done, assuming an explicit plane-wave form for the wavefunction where the spatial extent of a wavefront of constant phase is infinite. Two other specific cases for different source intensity distributions and individual neutron waveforms are summarized below.

Note that, for both of these examples, it is assumed that either the wavevector of the plane wave or the mean wavevector of the packet is always directed from a given point on the source line towards the origin (*x*, *y*) = (0, 0) of the laboratory reference frame. This should be a good approximation for the relatively small angles typically involved (*i.e.* where the small-angle approximation that sinθ ≃ θ is valid). And even though some plane-wave or packet mean wavevectors may be directed at locations other than the origin midway between points A_1_ and A_2_, a sufficiently balanced sampling of neutrons originating from various points along the source line should be ensured in performing the integration over possible trajectories.

Finally, we outline the derivation of two of the principal equations appearing in Section 7 of the main text, namely equations (18) and (20).

### b1. Plane waves, Gaussian source, leading to equation (18) in Section 7 of the main text

Given the plane wavefunction Ψ(*x*, *y*) = *C*_PW_exp(*ik_x_x*) × exp(*ik_y_y*) and a Gaussian source, the mutual coherence function can be written, beginning with equation (13) for the mutual coherence function, as

where *C*_PW_, *C*_GS_ and *C*_FTGS_ are normalization constants for the plane wave, Gaussian distribution and Fourier transform, respectively, and *C*_ΠPWGS_ is their product. Since

(the same product of constants occurs whether a Fourier transform or a simple integration of a Gaussian is performed), the complex degree of coherence [equation (14)] is then

### b2. Wave packet, Gaussian source, leading to equation (20) in Section 7 of the main text

Given the Gaussian wave packet Ψ(*x*, *y*) = *C*_WP_exp[−*y* ^2^/(2  )]exp(*ik*_M*x*_ *x*)exp(*ik*_M*y*_ *y*) and a Gaussian source, beginning again with equation (13) for the mutual coherence function,

where C_WP_, C_GS_ and C_FTGS_ are normalization constants for the wave packet, Gaussian distribution, and Fourier transform, respectively, and *C*_ΠWPGS_ is their product. Since

(the same product of constants occurs whether a Fourier transform or simple integration of a Gaussian is performed), then

which is the same as the result obtained in the previous example for the plane wavefunction.

### b3. Glossary of variables for Appendix *B* [App appb] and Section 7

Δ*r*_T_ = transverse coherence – defined as the spatial width, in general, over which the wavefronts of the wave packet do not fall out of phase by more than a certain specified amount – for the particular wave packets of Gaussian form discussed in Section 7 and Appendix *B* [App appb], Δ*r*_T_ is taken to be the standard deviation of the distribution σ_*y*WPα⊥_ as defined below

*V* = ‘fringe’ visibility

Ψ_D_ = resultant neutron wavefunction at a point D on the detector plane

A_1_ and A_2_ = apertures acting as secondary source points

*r*_1_ and *r*_2_ = distances from source point to apertures A_1_ and A_2_, respectively

^Σ^ *I*_D_ = net intensity at detector D

Γ_A1A2_ = mutual coherence function

γ_A1A2_ = normalized mutual coherence function or the complex degree of coherence

*k* = 2π/λ, where λ is the neutron wavelength

*k_x_* and *k_y_* = Cartesian components of the neutron wavevector **k**

2*s* = line source width extending from *y* = −*s* to *y* = +*s*

2*a* = distance between aperture points A_1_ and A_2_ along the *y* axis

*l* = distance between source line and aperture line

θ_1_ and θ_2_ = angles corresponding to the positions A_1_ and A_2_, respectively, on the *y* axis at +*a* and −*a*, where the wavefunctions are evaluated, which specify the neutron wavevector direction

*Y* = position along the vertical line source axis

*Terms associated with a Gaussian extended source distribution (applicable to either plane-wave or wave packet model)*

σ_S_ = the standard deviation of the Gaussian source intensity distribution representing the width of the source line along the *y* axis

σ_*k*_ = the standard deviation of a Gaussian distribution of *y* components of wavevectors (each wavevector corresponding either to a plane-wave wavevector or a packet mean wavevector) emanating from different source points (σ_*k*_ = 1/ σ_S_)

*Terminology specifically associated with plane-wave model*

*k*_⊥_ = *y* component of a plane-wave wavevector (the *y* axis is perpendicular to the nominal or average beam propagation direction along the *x* axis) for a plane wave emanating from a given point on an extended source line

σ_*k*⊥_ = standard deviation of a Gaussian distribution of the *y* components of the plane-wave wavevectors in a beam composed of plane waves emanating from an extended source line

*C*_PW_, *C*_GS_ and *C*_FTGS_ are normalization constants for the plane wave, Gaussian distribution and Fourier transform, respectively, and C_ΠPWGS_ is their product

*Terminology specifically associated with wave packet model*

σ_*y*WP⊥_ = standard deviation of the width of a packet in real space [as appears in equation (7)]

**k**_M_ = packet mean wavevector

*k*_M*x*_ and *k*_M*y*_ = *x* and *y* components of the packet mean wavevector **k**_M_, respectively

*k*_M⊥_ = *y* component of the packet mean wavevector (the *y* axis is perpendicular to the nominal or average beam propagation direction along the *x* axis) for a packet emanating from a given point on an extended source line

σ_*k*M⊥_ = standard deviation of a Gaussian distribution of the *y* components of the packet mean wavevectors in a beam composed of wave packets emanating from an extended source line

*C*_WP_, *C*_GS_ and *C*_FTGS_ are normalization constants for the wave packet, Gaussian distribution and Fourier transform, respectively, and *C*_ΠWPGS_ is their product
